# Multivalent lipid targeting by the calcium-independent C2A domain of synaptotagmin-like protein 4/granuphilin

**DOI:** 10.1074/jbc.RA120.014618

**Published:** 2020-12-10

**Authors:** Aml A. Alnaas, Abena Watson-Siriboe, Sherleen Tran, Mikias Negussie, Jack A. Henderson, J. Ryan Osterberg, Nara L. Chon, Beckston M. Harrott, Julianna Oviedo, Tatyana Lyakhova, Cole Michel, Nichole Reisdorph, Richard Reisdorph, Colin T. Shearn, Hai Lin, Jefferson D. Knight

**Affiliations:** 1 Department of Chemistry, University of Colorado Denver, Denver, Colorado, USA; 2 Department of Pharmaceutical Sciences, School of Medicine, University of Colorado Anschutz Medical Campus, Aurora, Colorado, USA; 3 Department of Pediatrics, Division of Pediatric Gastroenterology, Hepatology and Nutrition, School of Medicine, University of Colorado Anschutz Medical Campus, Aurora, Colorado, USA

**Keywords:** Slp4, granuphilin, C2 domain, membrane binding, electrostatics, insulin secretion, MIN6, ACN, acetonitrile, COM, center of mass, dansyl-PE, N-[5-dimethylamino)-naphthalene-1-sulfonyl]-1,2-dipalmitoyl-*sn*-glycero-3-phosphoethanolamine, IP_3_, inositol-(1,4,5)-trisphosphate, MD, molecular dynamics, PA, phosphatidic acid, PC, phosphatidylcholine, PDB, protein data bank, PI, phosphatidylinositol, PIP_2_, phosphatidylinositol-(4,5)-bisphosphate, PM, plasma membrane, PO_4_, phosphate, PS, phosphatidylserine, Slp-4, synaptotagmin-like protein 4

## Abstract

Synaptotagmin-like protein 4 (Slp-4), also known as granuphilin, is a Rab effector responsible for docking secretory vesicles to the plasma membrane before exocytosis. Slp-4 binds vesicular Rab proteins via an N-terminal Slp homology domain, interacts with plasma membrane SNARE complex proteins via a central linker region, and contains tandem C-terminal C2 domains (C2A and C2B) with affinity for phosphatidylinositol-(4,5)-bisphosphate (PIP_2_). The Slp-4 C2A domain binds with low nanomolar apparent affinity to PIP_2_ in lipid vesicles that also contain background anionic lipids such as phosphatidylserine but much weaker when either the background anionic lipids or PIP_2_ is removed. Through computational and experimental approaches, we show that this high-affinity membrane binding arises from concerted interaction at multiple sites on the C2A domain. In addition to a conserved PIP_2_-selective lysine cluster, a larger cationic surface surrounding the cluster contributes substantially to the affinity for physiologically relevant lipid compositions. Although the K398A mutation in the lysine cluster blocks PIP_2_ binding, this mutated protein domain retains the ability to bind physiological membranes in both a liposome-binding assay and MIN6 cells. Molecular dynamics simulations indicate several conformationally flexible loops that contribute to the nonspecific cationic surface. We also identify and characterize a covalently modified variant that arises through reactivity of the PIP_2_-binding lysine cluster with endogenous bacterial compounds and binds weakly to membranes. Overall, multivalent lipid binding by the Slp-4 C2A domain provides selective recognition and high-affinity docking of large dense core secretory vesicles to the plasma membrane.

Synaptotagmin-like protein 4 (Slp-4), also known as granuphilin, plays an important role in the trafficking and docking of insulin and other large dense core secretory vesicles to the plasma membrane (PM) before exocytosis (1). Two seemingly contradictory effects have been observed upon overexpression of Slp-4 in insulin-secreting MIN6 and INS-1 cell lines: (i) an increased number density of secretory granules docked to the PM before stimulation (2, 3), but (ii) decreased efficiency of secretion and a decreased total amount of insulin secreted upon stimulation with KCl or glucose (3, 4). Inversely, KO or knockdown of Slp-4 enhances insulin secretion (5, 6). The precise intermolecular interactions that give rise to these effects are not yet clear.

Slp-4 is a Rab effector protein that targets secretory vesicles via interaction of its N-terminal Slp homology domain with vesicular Rab27a, for which it recognizes both GDP- and GTP-bound forms (7). The central linker region of Slp-4 interacts with PM SNARE complex proteins (8, 9, 10, 11). The protein's tandem C-terminal C2 domains (C2A and C2B) bind anionic PM lipids including phosphatidylinositol-(4,5)-bisphosphate (PIP_2_) in a Ca^2+^-independent manner (12, 13). The interactions between Slp family members and Rab proteins are well studied; for example, point mutations in the Slp-4 homology domain that prevent binding to Rab27 are known (3, 7, 12, 14). However, the interactions that allow Slp-4 to bridge to the PM, including those of the C2 domains, are less thoroughly characterized.

C2 domains are the second-largest family of membrane-targeting protein domains in mammals and are found in a wide variety of signaling and membrane-trafficking proteins, including PKC and synaptotagmins (15, 16). Although several well-studied C2 domains are recruited to membrane surfaces through Ca^2+^ binding, and PIP_2_ enhances membrane affinity for some of them, many other C2 domains, including those of Slp-4, do not bind Ca^2+^ (16, 17). The membrane binding mechanisms of the Ca^2+^-independent C2 domains are generally less well understood.

The available crystal structure of the Slp-4 C2A domain (protein data bank [PDB]: 3FDW) aligns well with other type I topology C2 domains, including those from synaptotagmin (15) (Fig. 1). It is missing four of the five conserved Asp residues in the β2–β3 and β6–β7 loops that coordinate Ca^2+^ in Ca^2+^-dependent C2 domains (15, 18). However, it contains a PIP_2_-binding sequence including a cluster of lysine residues on the β3 and β4 strands, also termed the polybasic region, which is found in PIP_2_-sensitive, Ca^2+^-dependent type I topology C2 domains such as PKCα, the C2B domain of synaptotagmin-1, and the C2A domains of rabphilins (16). The PIP_2_ binding site of the Slp-4 C2A domain was previously reported to center on this cluster (19). Notably, a large region around the cluster contains other basic residues that impart a positively charged electrostatic surface (Fig. 1).

**Figure 1:**
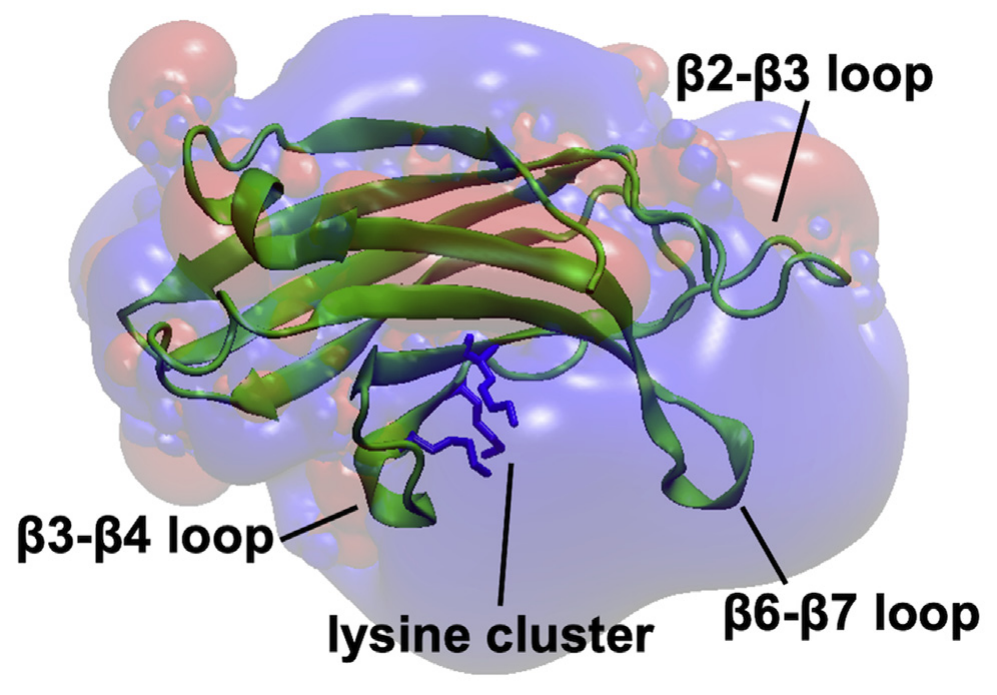
**Slp-4 C2A domain structure and electrostatic surface.** The locations of important loops and the PIP_2_-binding lysine cluster are shown, along with an electrostatic surface map showing the large positive (*blue*) surface surrounding the lysine cluster. PIP_2_, phosphatidylinositol-(4,5)-bisphosphate; Slp-4, synaptotagmin-like protein 4.

The Slp-4 C2A domain plays a critical role in targeting its parent protein to the PM in secretory cells. Overexpression of a ΔC2AB mutant leads to fewer vesicles docked to the PM than that of the full-length Slp-4, and the mutant protein localizes to the cytosol and interior vesicles rather than to docked vesicles (6, 12). However, deleting only the C2B domain has little effect; notably, the Slp-4b splice variant lacks a C2B domain but has similar function compared with the full-length Slp-4a version (4, 6, 10). Our group has previously shown that the Slp-4 C2A domain binds with apparent low nanomolar affinity to PIP_2_ in liposomes with physiological lipid compositions, whereas the affinity of the C2B domain toward the same liposomes is approximately 50-fold weaker (20). Therefore, the C2A domain is likely the most important mediator of the Slp-4 PM interaction.

Multivalent protein-membrane interactions are abundant among membrane-targeting proteins and have been suggested to constitute the principal mechanism for achieving specificity in targeting particular subcellular membranes (16, 21). Interestingly, the Slp-4 C2A domain binds with only modest (∼1 μM) affinity to soluble PIP_2_ or to phosphatidylcholine (PC)/PIP_2_ nanodiscs (19), which is ∼100-fold weaker than its apparent affinity for PIP_2_ in liposomes with physiological lipid composition (20). This discrepancy suggests that besides the PIP_2_-binding site, there must be other lipid contacts that stabilize the association of the Slp-4 C2A domain to lipid compositions resembling the PM.

In this work, we have sought to identify how background anionic PM lipids, such as phosphatidylserine (PS) and phosphatidylinositol (PI), work in conjunction with PIP_2_ to mediate strong membrane binding of the Slp-4 C2A domain. Using purified C2A domains and synthetic liposomes with controlled lipid compositions, we have tested the contributions of PIP_2_ and other anionic lipids to liposome affinity as well as kinetic on- and off-rates. As a complement to these experiments, we have used computational docking and molecular dynamics (MD) simulations to predict which regions of the polycationic protein surface interact with each target lipid head group or insert into the hydrophobic interior of a lipid bilayer. Finally, we have tested the effect of single and multiple point mutations on the C2A domain's membrane binding in a liposome-binding assay, in MD simulations, and in live cells. To our knowledge, this is the first study to apply this range of approaches to investigate the membrane binding mechanism of a Ca^2+^-independent, PIP_2_-targeting C2 domain. Overall, we find that while binding to PIP_2_ occurs primarily through the previously identified lysine cluster (19), the protein retains the ability to bind physiological membranes even when PIP_2_ binding is disrupted by mutation. A significant portion of the overall free energy of membrane binding comes from interaction with anionic background lipids via a large surface surrounding the lysine cluster and encompassing multiple loop regions. Furthermore, we show for the first time that the conserved lysine cluster is susceptible to covalent modification by carbonyl-containing compounds during bacterial protein expression, and we characterize the effect of this modification on protein-membrane binding.

## Results

### Strong Slp-4 C2A membrane binding requires PS and PIP_2_

The C2A domain of Slp-4 is known to drive this protein's ability to bind cellular PMs primarily via interaction with PIP_2_ (12, 13). However, the protein retains an affinity for membranes containing the anionic lipids PS and/or PI, even in the absence of PIP_2_ (20). Here, we set out to discern the contributions of PIP_2_ and background anionic lipid binding to its strong affinity for physiological lipid membranes, including determining residues that drive binding to anionic background lipids. To do this, we first measured interactions between purified protein domains and liposomes of defined lipid composition (Table 1).

**Table 1:** Lipid compositions used in this study

Name	Target membrane lipid compositions (mol %)							
	PE	PC	PS	PI	PIP_2_	SM	CH	Dansyl-PE^a^
PM	27.9	10.6	21.3	3.6	2.0	4.6	25.0	5.0
PM(-)PIP_2_	27.9	12.6	21.3	3.6	-	4.6	25.0	5.0
PM(-)PS/PI	46.8	16.6	-	-	2.0	4.6	25.0	5.0
PM[4%PIP_2_]	27.9	8.6	21.3	3.6	4.0	4.6	25.0	5.0

CH, cholesterol; PC, phosphatidylcholine; PE, phosphatidylethanolamine; PI, phosphatidylinositol; PIP_2_, phosphatidylinositol-(4,5)-bisphosphate; PS, phosphatidylserine; SM, sphingomyelin.

a For unlabeled liposomes used in stopped-flow off-rate measurements, the compositions were the same except that dansyl-PE was replaced with PE.

**Figure 2:**
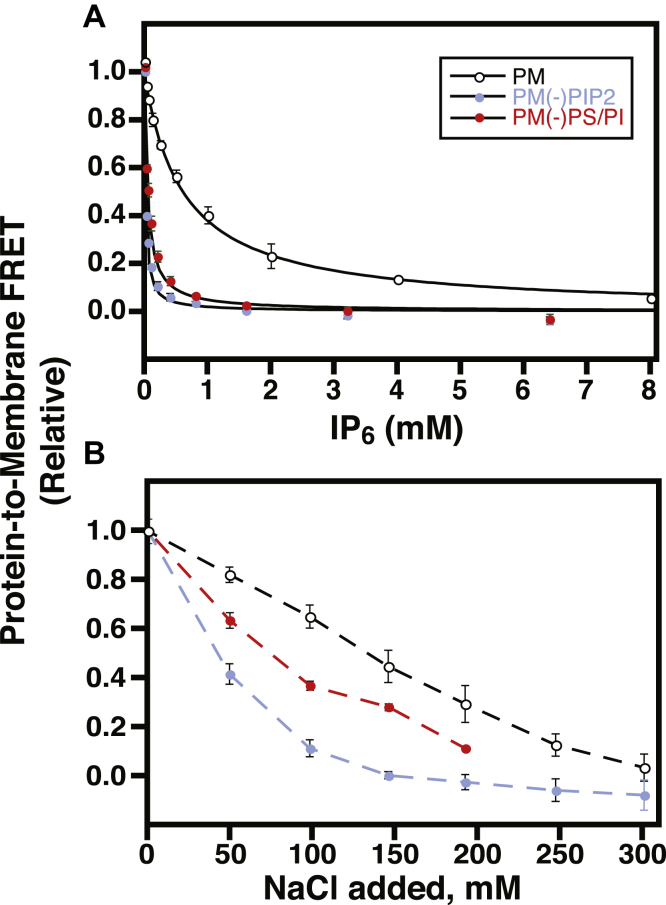
**Lipid dependence of Slp-4 C2A domain membrane binding and anion inhibition.***A*, IP_6_ competition titrations for Slp-4 C2A initially bound to liposomes of the indicated compositions. Best-fit curves to a competitive inhibition binding model (Equation 2) are shown; IC_50_ values are given in Table 2. *B*, NaCl screening titrations. *Dashed lines* guide the eye. Error bars are SD of 3 independent replicate titrations and where not visible are smaller than the data points. Colors represent different liposome compositions (Table 1): *black*, PM; *blue*, PM(-)PIP_2_; *red*, PM(-)PS/PI. PI, phosphatidylinositol; PIP_2_, phosphatidylinositol-(4,5)-bisphosphate; PM, plasma membrane; PS, phosphatidylserine; Slp-4, synaptotagmin-like protein 4.

Although the Slp-4 C2A domain has a strong affinity for liposomes with a lipid composition approximating the PM interior leaflet, removal of either PIP_2_ or background anionic lipids (PS and PI) decreases its affinity by an order of magnitude (Fig. 2*A*). We measured the relative affinities of the C2A domain for various synthetic liposomes containing N-[5-(dimethylamino)-naphthalene-1-sulfonyl]-1,2-dipalmitoyl-*sn*-glycero-3-phosphoethanolamine (dansyl-PE) lipids by measuring tryptophan-to-dansyl FRET of the lipid-bound Slp-4 C2A domain while titrating with the soluble inhibitor d-*myo*-inositol-(1,2,3,4,5,6)-hexakisphosphate (IP_6_). We have previously demonstrated that this assay reports accurately on the membrane affinity of the protein (20). For a given concentration of liposomes and protein, the concentration of inhibitor required to remove 50% of the protein initially bound to the membrane (IC_50_) is approximately proportional to the mole-fraction equilibrium constant *K*_x_ for partitioning onto the liposome surface (Equation 2, see Experimental materials and methods). When liposomes were used approximating the PM inner leaflet lipid composition (Table 1), the IC_50_ of IP_6_ was 620 ± 80 μM, corresponding to a binding constant of (30 ± 5) × 10^7^ (Fig. 2*A*, Table 2). Consistent with our previous report (20), removal of PIP_2_ from the liposome lipid composition [PM(-)PIP_2_] decreased the IC_50_ and the membrane partitioning coefficient by a factor of ∼30 (Table 2). Here, we also show that removal of background anionic lipids PS and PI while maintaining 2% PIP_2_ in the liposomes [PM(-)PS/PI] also significantly decreased the IC_50_, reflecting a ∼12-fold decrease in the affinity (Fig. 2*A*; Table 2). The membrane binding is primarily electrostatic, as binding to all three liposome compositions could be screened by NaCl at 2–3× physiological ionic strength (Fig. 2*B*). Increased salt concentration more efficiently screened binding to liposomes lacking PIP_2_ or background anionic lipids, relative to liposomes containing both. Thus, physiologically relevant levels of both PIP_2_ and background anionic lipids make important contributions to membrane binding for this protein.

**Table 2:** Equilibrium IP_6_ titration parameters of WT Slp-4 C2A domain

Liposome composition (see Table 1)	IC_50_ for IP_6_ (μM)^a^	*K*_x_ × 10^−6^^b^	Δ*G*° for binding (kcal mol^−1^)^c^
PM	620 ± 80	300 ± 50	−11.6 ± 0.1
PM(-)PIP_2_	19 ± 1	8 ± 1	−9.4 ± 0.1
PM(-)PS/PI	51 ± 6	24 ± 4	−10.1 ± 0.1

PI, phosphatidylinositol; PIP_2_, phosphatidylinositol-(4,5)-bisphosphate; PM, plasma membrane; PS, phosphatidylserine; Slp-4, synaptotagmin-like protein 4.

a Calculated using Equation 1.

b Calculated using Equation 2.

c Calculated using Equation 3.

**Figure 3:**
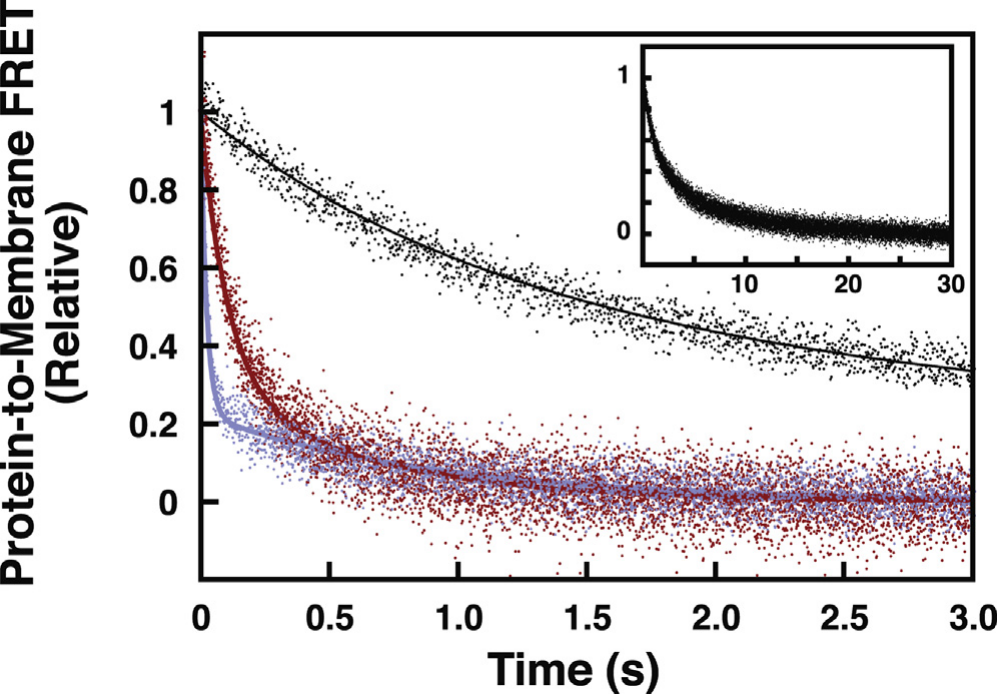
**Lipid composition dependence of Slp-4 C2A domain dissociation kinetics.** Representative dissociation curves are shown from PM (*black*), PM(-)PIP_2_ (*blue*), and PM(-)PS/PI (*red*) liposomes (see Table 1 for lipid compositions). *Inset*: full time course of the PM dissociation curve. PI, phosphatidylinositol; PIP_2_, phosphatidylinositol-(4,5)-bisphosphate; PM, plasma membrane; PS, phosphatidylserine; Slp-4, synaptotagmin-like protein 4.

Stopped-flow kinetic measurements of Slp-4 C2A domain liposome dissociation also show a pronounced dependence on both PIP_2_ and background anionic lipids. We previously reported that the removal of PIP_2_ increases the membrane dissociation rate of the domain while having no significant effect on the association rate (20). Here, we show that removal of background anionic lipids also significantly increased the kinetics of dissociation (Fig. 3; Table 3, *k*_off_), while having little effect on the association rate constant (Table 3, *k*_on,x_). The strong dependence of dissociation rates on both PIP_2_ and background anionic lipids indicates that both species contribute significantly to the thermodynamic stability of the membrane-bound state. Furthermore, the affinity differences among the various lipid compositions tested are due almost entirely to the differences in the off-rate. Dissociation from all liposome compositions was revealed to be biexponential, suggesting the presence of multiple membrane-bound states, which could reflect populations associated with different numbers of bound lipids. This feature was not apparent in our previous report because of a smaller signal-to-noise ratio (20).

**Table 3:** Stopped-flow kinetic parameters of WT Slp-4 C2A domain

Liposome composition (see Table 1)	*k*_obs_ (s^−1^)	*k*_on,x_ (s^−1^) ×10^−6^^a^	*k*_off_ (s^−1^)
PM	30 ± 12	43 ± 18	1.0 ± 0.3 (35 ± 18% amp) 0.14 ± 0.03 (65 ± 18% amp)
PM(-)PIP_2_	90 ± 50	90 ± 60	21 ± 13 (66 ± 13% amp) 2.6 ± 1.3 (34 ± 13% amp)
PM(-)PS/PI	55 ± 15	73 ± 23	8.0 ± 1.5 (60 ± 15% amp) 1.3 ± 0.4 (40 ± 15% amp)
PM/4%PIP_2_	47 ± 3	69 ± 8	0.43 ± 0.14 (27 ± 19% amp) 0.08 ± 0.05 (73 ± 19% amp)

PI, phosphatidylinositol; PIP_2_, phosphatidylinositol-(4,5)-bisphosphate; PM, plasma membrane; PS, phosphatidylserine; Slp-4, synaptotagmin-like protein 4.

a Calculated using Equation 7.

Increasing the PIP_2_ concentration in the PM liposome composition from 2% to 4% further slowed dissociation by about a factor of 2 relative to the PM (Table 3). This factor is much smaller than the ∼10-fold difference in the off-rate comparing PM(-)PIP_2_ to PM compositions, suggesting that the increased PIP_2_ concentration allows engagement of weaker, secondary binding site(s) on the protein. This secondary binding appears to be nonselective, as phosphatidic acid (PA) had a similar effect: dissociation from liposomes containing 2% PIP_2_ and 2% PA proceeded on a timescale comparable with PM/4% PIP_2_ (Fig. S1; Table S1). In contrast, the primary PIP_2_ binding is highly selective for PIP_2_ over PA, as dissociation from liposomes containing 2% PA but not PIP_2_ was much faster than from PM liposomes [Fig. S1 and Table S1, compare PM(-)PIP_2_(+)PA to PM]. Overall, the results of kinetic experiments confirm that this C2 domain's strong membrane affinity relies on the presence of both PIP_2_ and background anionic lipids.

### Predicting binding sites for anionic ligands using computational modeling

**Figure 4:**
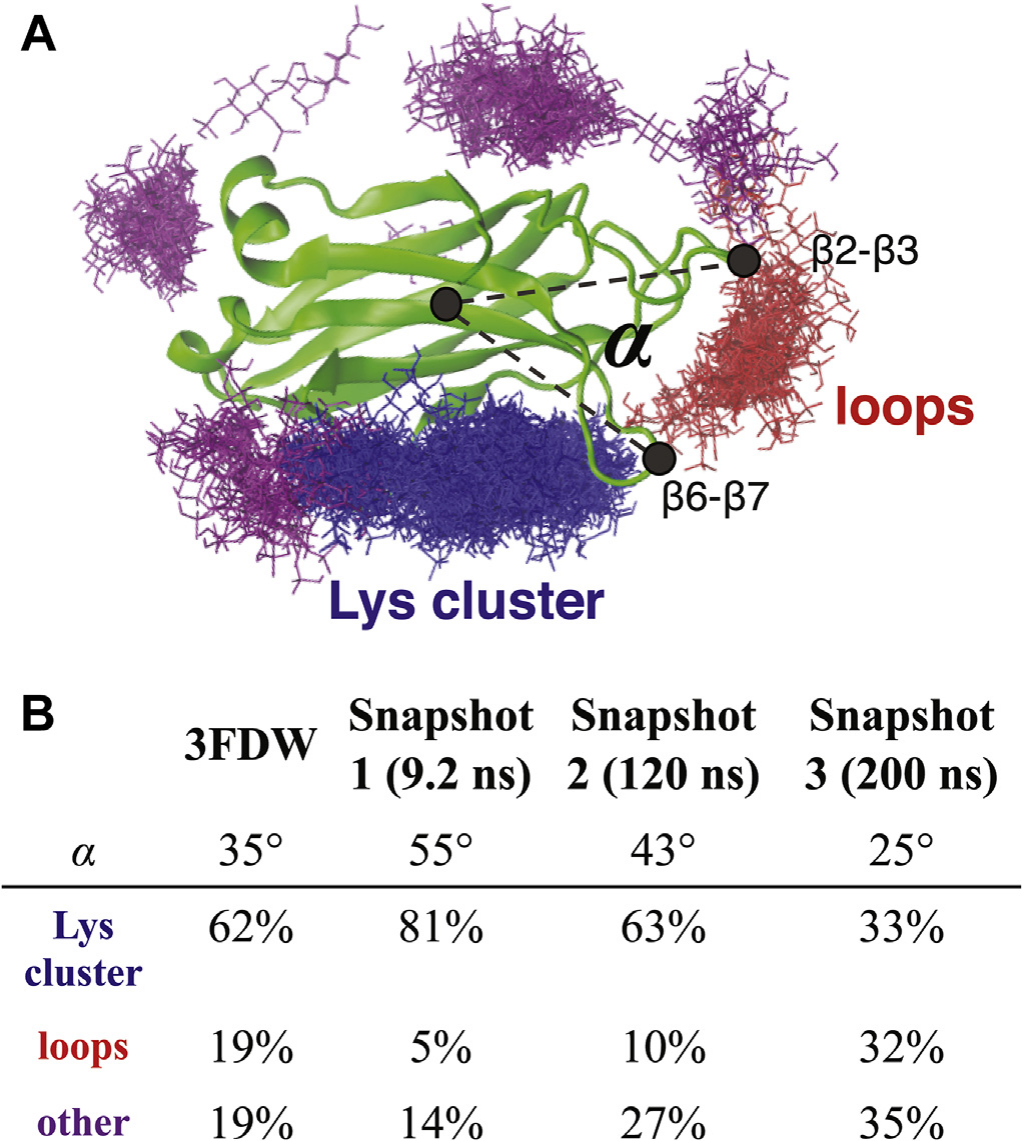
**Docking calculations with Slp-4 C2A domain**. *A*, representative clustering of docked IP_3_ ligands following Flexidock calculations. A total of 510 starting configurations were modeled, and all of the final ligand positions are shown overlaid in a *stick format*, colored by location cluster. *Gray dashed lines* define the angle *α* between the β2–β3 and β6–β7 loops. *B*, tabulation of *α* angle and ligand clustering for four similar sets of Flexidock calculations, each using a different starting protein conformation from the crystal structure (3FDW) or from the indicated time point of the standalone MD simulation (Movie S1). Slp-4, synaptotagmin-like protein 4.

To predict which regions of the Slp-4 C2A domain bind PIP_2_ or other anionic ligands, we simulated inositol-(1,4,5)-trisphosphate (IP_3_), a soluble PIP_2_ analogue, docking to the protein domain using a molecular docking algorithm. The first simulation included 510 separate docking calculations for IP_3_ molecules placed at a library of positions around the published protein structure (PDB: 3FDW). As expected, the majority of these calculations showed docking to the known PIP_2_ binding site, which is centered on the cluster of conserved lysine residues (Lys398, Lys410, and Lys412) in the β3 and β4 strands (Fig. 4, Lys cluster) (16, 19).

Several other clusters emerged from these calculations as secondary docking sites for IP_3_, suggesting they could represent nonspecific binding sites for anionic ligands. The largest of these clusters was near the tips of the β2–β3 and β6–β7 loops, which are structurally homologous to Ca^2+^-binding loops 1 and 3 in Ca^2+^-sensitive C2 domains (Fig. 4, loops) (15). These loop regions are known to be dynamic in Ca^2+^-free C2 domains (22, 23); therefore, we conducted a 200-ns MD simulation of the protein domain alone in a physiological salt solution at pH 7 (Supporting information, Movie S1). As expected, the loop regions showed conformational fluctuations during this simulation while the core of the domain remained intact. In particular, the β6–β7 loop moved closer to the β2–β3 loop during the simulation (Fig. S2); *i.e.*, the angle α shown in Figure 4*A* and Figure S2A decreased over time after an initial rapid increase.

Three snapshots from this simulation with varying α angles were chosen as templates for additional IP_3_ docking calculations. The results of these calculations are tabulated in Figure 4*B* alongside those of the original calculation based on the crystal structure. Docking to the loops site correlated strongly with α: as α decreased during the MD simulation, the percentage of docking events to the primary PIP_2_-binding site decreased, whereas docking to the tips of the loops increased. This result suggests that proximity between these two loops, both of which contain basic residues, improves affinity toward anionic ligands at this site. Further docking calculations using protein variants with mutations in the primary PIP_2_ binding site shifted IP_3_ binding further toward the loops site, consistent with the hypothesis that this is a preferred secondary binding site for anionic ligands (Table S2).

### Mapping protein–lipid contacts from MD simulations

**Figure 5:**
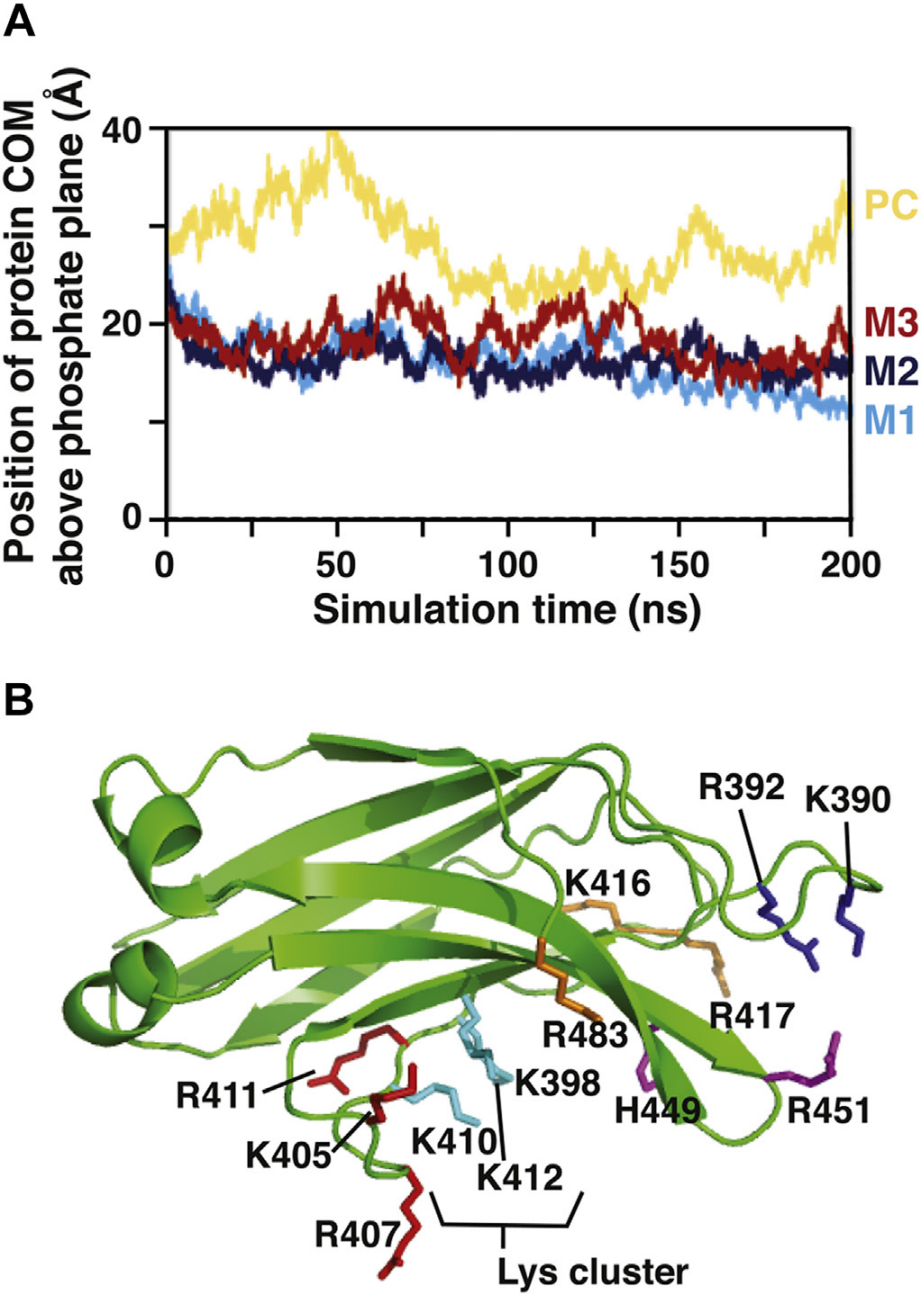
**Mapping protein-membrane contacts from MD simulations.***A*, the height of the protein center of mass (COM) above the phosphate plane of the membrane during each simulation. *Yellow*: PC control; *light blue*: model 1 (M1); *dark blue*: model 2 (M2); *red*: model 3 (M3). *B*, residues that make significant ionic contact with PS or PIP_2_ in at least one of the simulations are labeled. *Cyan*: conserved lysine cluster; *blue*: β2–β3 loop; *red*: β3–β4 loop, and β4 strand; *purple*: β6–β7 loop; *orange*: β4–β5 loop and C-terminus. PC, phosphatidylcholine; PIP_2_, phosphatidylinositol-(4,5)-bisphosphate; PS, phosphatidylserine.

MD simulations of the Slp-4 C2A domain docking to anionic membranes indicate that multiple regions of the protein domain, including the β2–β3 and β6–β7 loops, make electrostatic and/or hydrophobic contacts with the membrane. To illuminate what residues might make key contacts with target lipids, we performed three parallel MD simulations of the Slp-4 C2A domain binding to lipid bilayers composed of PC, PS, and PIP_2_ (Fig. S3; Movies S2–S4). The three simulations differed in the initial placement of the protein with respect to the two PIP_2_ molecules embedded in the target membrane leaflet. In models 1 and 2, the protein was placed midway between the two PIP_2_ molecules, with its long axis oriented perpendicular to (model 1) or along (model 2) the line between the PIP_2_ molecules, whereas in model 3 the protein was placed with its lysine cluster directly above one of the PIP_2_ molecules. In all three simulations, the protein rapidly made contact with the lipids immediately below it within the first 20 ns, after which the center-of-mass (COM) position of the protein remained relatively constant relative to the membrane phosphate (PO_4_) plane (Fig. 5*A*). Because of this rapid membrane association and the initial configurations of each system, the lysine cluster only interacted with PIP_2_ in model 3; in models 1 and 2, the PIP_2_ molecules interacted with residues at the periphery of membrane contact. This result suggests that membrane rearrangements that allow PIP_2_ insertion into its primary binding site occur on a timescale inaccessible in these simulations.

A control simulation was also performed with the protein initially positioned above a PC bilayer (Fig. S3; Movie S5). In this simulation, the protein initially drifted away from the membrane before making transient contact with the polar head group region via the β3–β4 loop (Fig. 5*A*). We have reported previously that the Slp-4 C2A domain does not bind PC liposomes to a measurable extent using the protein-to-membrane FRET assay (20). The transient contact seen between ~75 ns and ~150 ns in the PC control simulation therefore likely reflects weak and nonspecific interactions under the relatively closely confined conditions of the simulation (Fig. 5*A*).

From the PC/PS/PIP_2_ simulation data, protein–lipid contacts were identified throughout the polycationic surface of the protein (Fig. 1). Residues making contact with PS and PIP_2_ included the two regions predicted from the IP_3_ docking calculations: the lysine cluster and the loops region (which includes the β2–β3 loop and the β6–β7 loop) (Fig. 5*B*; Table 4). In addition, basic residues in the β3–β4 loop near the lysine cluster made significant contact. The β2–β3 and β6–β7 loops remained much closer together (small α angle) throughout all of the simulations than in the crystal structure (Fig. S2, *B–C*). Residues that average ≥0.5 contacts with PS or PIP_2_ during the last 100 ns of at least one simulation are quantified in Table 4. These include Lys390 and Arg392 in the β2–β3 loop, several residues in the β3–β4 region, and His449 and Arg451 in the β6–β7 loop. Contacts for other basic residues from the MD simulations are listed in Table S3. Notably, Lys398 contributed centrally to PIP_2_ coordination in model 3 (in which the PIP_2_ was bound near the previously reported binding pocket) but did not coordinate PS or PIP_2_ in the other simulations. The lipid contacts of each residue are plotted as a function of simulation time in Figures S4 and S5.

**Table 4:** Electrostatic hydrophilic contacts

Location	Residue	PC control	Model 1		Model 2		Model 3	
		Average PC contact	Average PS contact	Average PIP_2_ contact	Average PS contact	Average PIP_2_ contact	Average PS contact	Average PIP_2_ contact
β2–β3 loop	K390	0	1.3 ± 0.8	0	0.3 ± 0.5	0.1 ± 0.0	0	0
	R392	0	1.1 ± 0.0	0	0.1 ± 0.3	1.0 ± 0.0	0.5 ± 0.7	0
β3–β4 loop	K405	1.8 ± 0.8	0	0	0	1.0 ± 0.1	0.3 ± 0.5	0
	R407	3.0 ± 0.7	0.1 ± 0.3	0	0.2 ± 0.4	1.0 ± 0.0	0.9 ± 0.5	0
Lys cluster	K398	0.3 ± 0.6	0	0	0	0	0.6 ± 0.5	1.0 ± 0.1
	K410	0.1 ± 0.3	0	0	0	0	0.4 ± 0.5	0
β4	R411	0.4 ± 0.7	1.6 ± 0.6	0	0.2 ± 0.4	0	0.7 ± 0.5	0
Lys cluster	K412	0.2 ± 0.4	0.8 ± 0.5	0	0.1 ± 0.3	0	0	1.0 ± 0.0
β4–β5 loop	K416	0	0.3 ± 0.5	0	0.5 ± 0.5	0	0	0
	R417	0.1 ± 0.4	0.2 ± 0.5	0	1.0 ± 0.6	0.1 ± 0.3	0.3 ± 0.5	0
β6–β7 loop	H449	0.1 ± 0.3	0	0	0.2 ± 0.4	0	0	0.9 ± 0.3
	R451	0	0	0	0.5 ± 0.5	0	0.5 ± 0.8	0
C-term	K483	0.2 ± 0.4	0	1.0 ± 0.2	0	0	0	0

PC, phosphatidylcholine; PIP_2_, phosphatidylinositol-(4,5)-bisphosphate; PS, phosphatidylserine.

The number of the indicated lipid molecules (±SD) contacted by each residue, averaged over the last 100 ns of simulations, is displayed for the PC control and PC/PS/PIP2 models 1, 2, and 3. Residues listed here had contact numbers ≥0.5 in any of the simulations; an extended list including all basic residues is given in Table S3. Contacts are defined as described in MD simulations and docking calculations.

Although the membrane contacts of this protein domain are predominantly ionic, there were also uncharged residues on the β3–β4 loop that made extensive contact with the membrane in the MD simulations, including the PC control. This loop, adjacent to the previously reported PIP_2_-binding site, showed considerable dynamics, inserting toward the lipid head group region upon membrane contact in the model 1 and PC control simulations (Fig. S2; Movies S2 and S5). The backbone of the β3–β4 loop in the region of Arg407, Gln408, and Gly409 penetrated at or near the depth of the lipid PO_4_ plane in each of the simulations (Table S4 and Fig. S6). The side chain of Gln408 was, at times, observed to insert below the PO_4_ plane in model 1 and to make H-bonds with ester carbonyl oxygens on PC (Fig. S7).

The only significant penetration observed into the nonpolar portion of the membrane was from the side chain of Phe452 on the β6–β7 loop. This residue had an average side chain insertion depth of 2.5 Å below the PO_4_ plane in model 1 (Table S4). Phe452 inserted near the PO_4_ plane depth in the other two simulations (Table S4; Fig. S6).

### Identifying membrane-binding residues via site-directed mutagenesis

**Figure 6:**
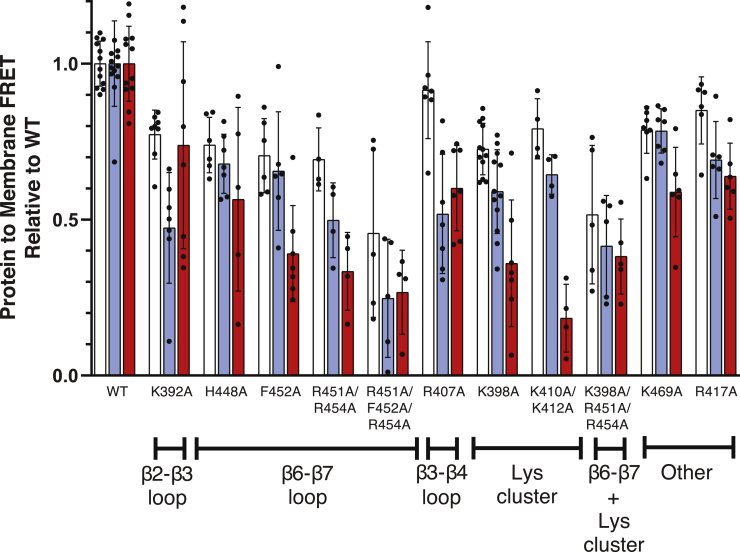
**Effects of mutations on Slp-4 C2A liposome interaction.** For each indicated mutant C2A domain (1 μM), Trp-to-dansyl FRET was measured upon addition of liposomes (65-μM total accessible lipid) as described in Experimental materials and methods. The extent of FRET was normalized to that of the WT protein domain for each liposome composition. *White*, PM; *blue*, PM(-)PIP_2_: *red*, PM(-)PS/PI (Table 1). Error bars are ±SD of ≥4 samples; individual data points are shown. PI, phosphatidylinositol; PIP_2_, phosphatidylinositol-(4,5)-bisphosphate; PM, plasma membrane; PS, phosphatidylserine; Slp-4, synaptotagmin-like protein 4.

To test experimentally for the functional importance of the membrane-contacting residues identified in the MD simulations, we selectively mutated residues to alanine, either individually or in combination. Liposome binding activity of each mutant was assessed by measuring the extent of protein-to-membrane FRET for each mutant upon addition of PM, PM(-)PIP_2_, and PM(-)PS/PI liposomes (Fig. 6). As expected, mutation of residues in the known PIP_2_-binding site (K398A and K410A/K412A) had significant negative effects on binding to PM(-)PS/PI liposomes, in which PIP_2_ is the only target lipid. However, these mutants retained >50% binding toward PM and PM(-)PIP_2_ liposomes, indicating that they are still capable of nonspecific electrostatic interactions. The greatest impacts on binding to full PM lipid compositions were found with two triple mutants: K398A/R451A/R454A and R451A/F452A/R454A; however, even these retained ∼50% of PM liposome binding relative to the WT domain under the conditions of the assay (Fig. 6).

The thermal folding stability of the mutated C2 domains was assessed using differential scanning fluorimetry (Fig. S8). All of the mutants included in Figure 6 had clear melting transitions with melting temperatures higher than those of WT, indicating that neutralization of positive charge and/or mutation of the surface-exposed Phe452 improved the thermodynamic folding stability of the domain. Another mutant, R411A, showed high initial intensity and no melting transition, indicating that the R411A mutant does not fold stably (Fig. S8). The H448A mutant also had a higher initial intensity, indicating some surface-exposed hydrophobic groups but retained a clear unfolding transition at a temperature similar to the WT domain.

**Figure 7:**
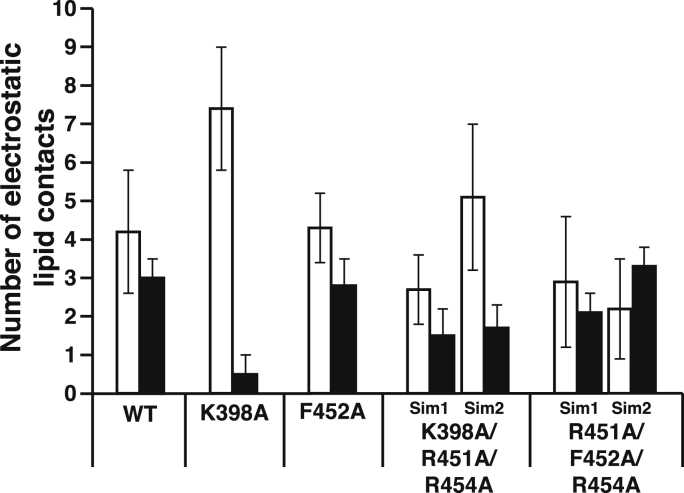
**Effects of mutations on Slp-4 C2A lipid binding from MD simulations.** The number of basic residues making electrostatic contact with PS (*open bars*) or PIP_2_ (*filled bars*) was calculated from the individual residue contacts as described in MD simulations and docking calculations, averaged (±SD) over the final 100 ns of simulation. For the K398A/R451A/R454A and R451A/F452A/R454A mutants, two simulations were conducted which differed only in the random seeds used to calculate initial velocities; data from both simulations for each mutant are shown (Sim1 and Sim2). A breakdown of PS and PIP_2_ contacts by residue for each simulation is given in Table S5. PIP_2_, phosphatidylinositol-(4,5)-bisphosphate; PS, phosphatidylserine; Slp-4, synaptotagmin-like protein 4.

To understand how these mutants retain interactions with PIP_2_ and PS, we conducted MD simulations of selected mutant domains binding to PC/PS/PIP_2_ bilayers, using a starting geometry identical to model 3 of the WT protein. The single K398A mutation in the PIP_2_-binding lysine cluster resulted in decreased PIP_2_ contact but increased PS contact relative to WT (Fig. 7). The single F452A mutation also did not change anionic lipid contacts appreciably, as expected. The triple mutants R451A/F452A/R454A and K398A/R451A/R454A had decreased anionic lipid contact relative to the WT domain (Fig. 7), particularly in the β6–β7 loop (Table S5). Nevertheless, even these triple mutants retained at least 50% of their anionic lipid contacts relative to those of WT in all simulations, consistent with results from the liposome binding assays (Fig. 6). For example, loss of Lys398 contact with anionic lipids in some simulations was compensated through increased PIP_2_ contact by Lys410 (Table S5). Repeat simulations (varying only by the seed in a random number generator) yielded somewhat different lipid binding contacts for the two triple mutants; however, they did not change the overall qualitative picture, reflecting the availability of a large number of basic residues on the lipid-binding surface.

### Slp-4 C2A membrane localization in MIN6 cells

**Figure 8:**
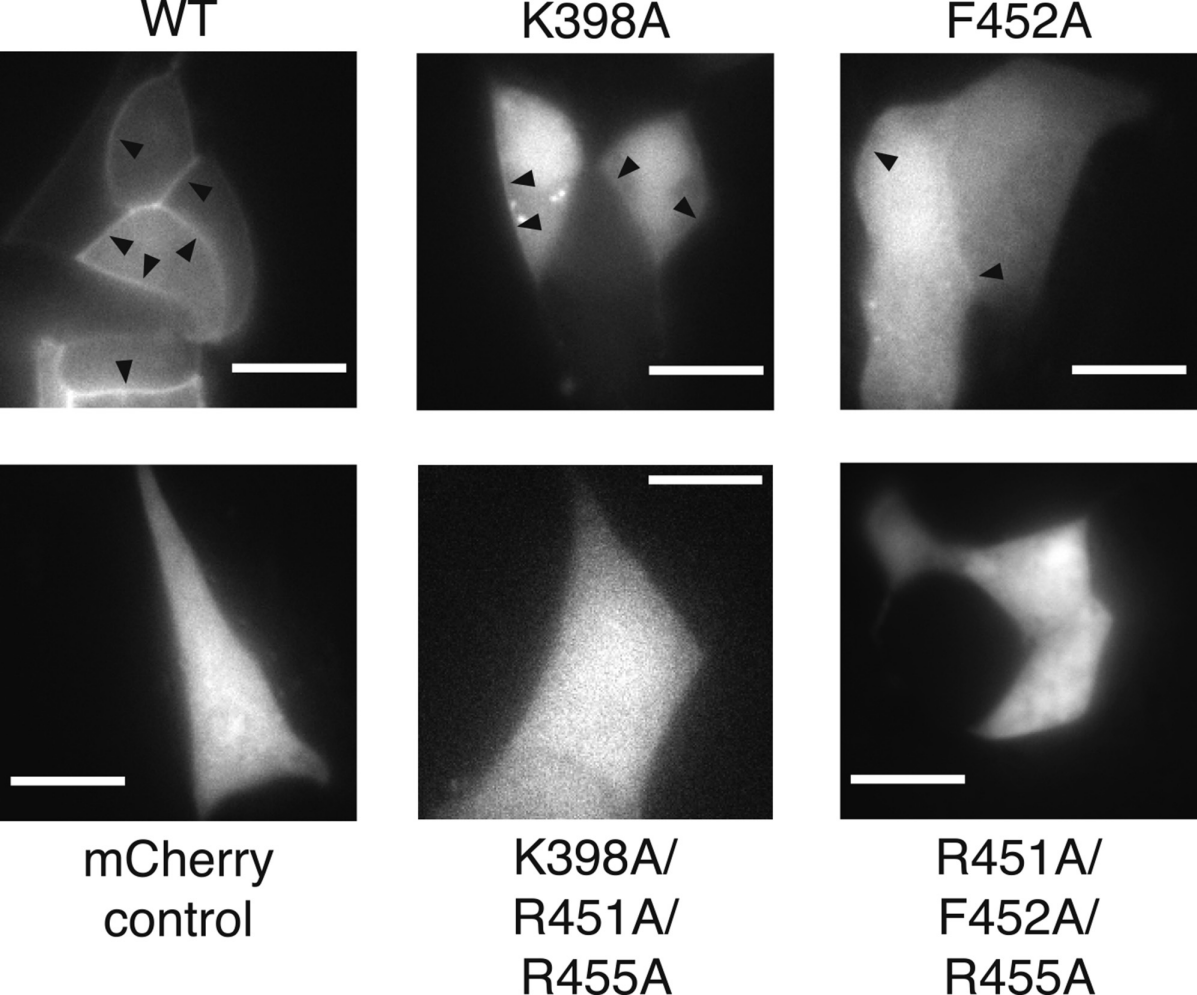
**Cellular localization of mCherry-Slp4 C2A domain and mutants**. Plasmids encoding mCherry alone (mCherry control), mCherry fused to the C2A domain of Slp-4 (WT), or the indicated mutants were transiently expressed in MIN6 cells. Representative fluorescence microscopy images are shown. *Arrowheads* indicate sites of membrane localization. Scale bars are 10 μm. Slp-4, synaptotagmin-like protein 4.

To test whether these selected mutations impact membrane binding in secretory cells, we transiently expressed WT or mutant Slp-4 C2A domains fused to mCherry in MIN6 cells and imaged the live cells using fluorescence microscopy. As expected, the WT C2A domain appeared to localize primarily to the cell periphery, whereas fluorescence of an mCherry control was evenly distributed throughout the cell (Fig. 8). The single mutants K398A and F452A each retained some membrane localization, although less than WT. These results show that Slp-4 C2A membrane binding in secretory cells is diminished but not eliminated when PIP_2_ binding is blocked (K398A) or hydrophobic insertion is eliminated (F452A). Membrane binding appeared to be nonexistent in the triple mutants, both of which were indistinguishable from the cytoplasmic mCherry control (Fig. 8). Thus, all of these mutations have a greater impact on Slp-4 C2A membrane binding in the environment of the cell than in the conditions used for our MD simulations and *in vitro* liposome-binding assay. However, the trend of decreased membrane binding in the triple versus single mutants remains consistent. These results, taken together, demonstrate that multiple regions of the Slp-4 C2A domain contribute to its strong affinity for physiological lipid membranes.

### Identification and characterization of bacterial modifications in the lysine cluster

**Figure 9:**
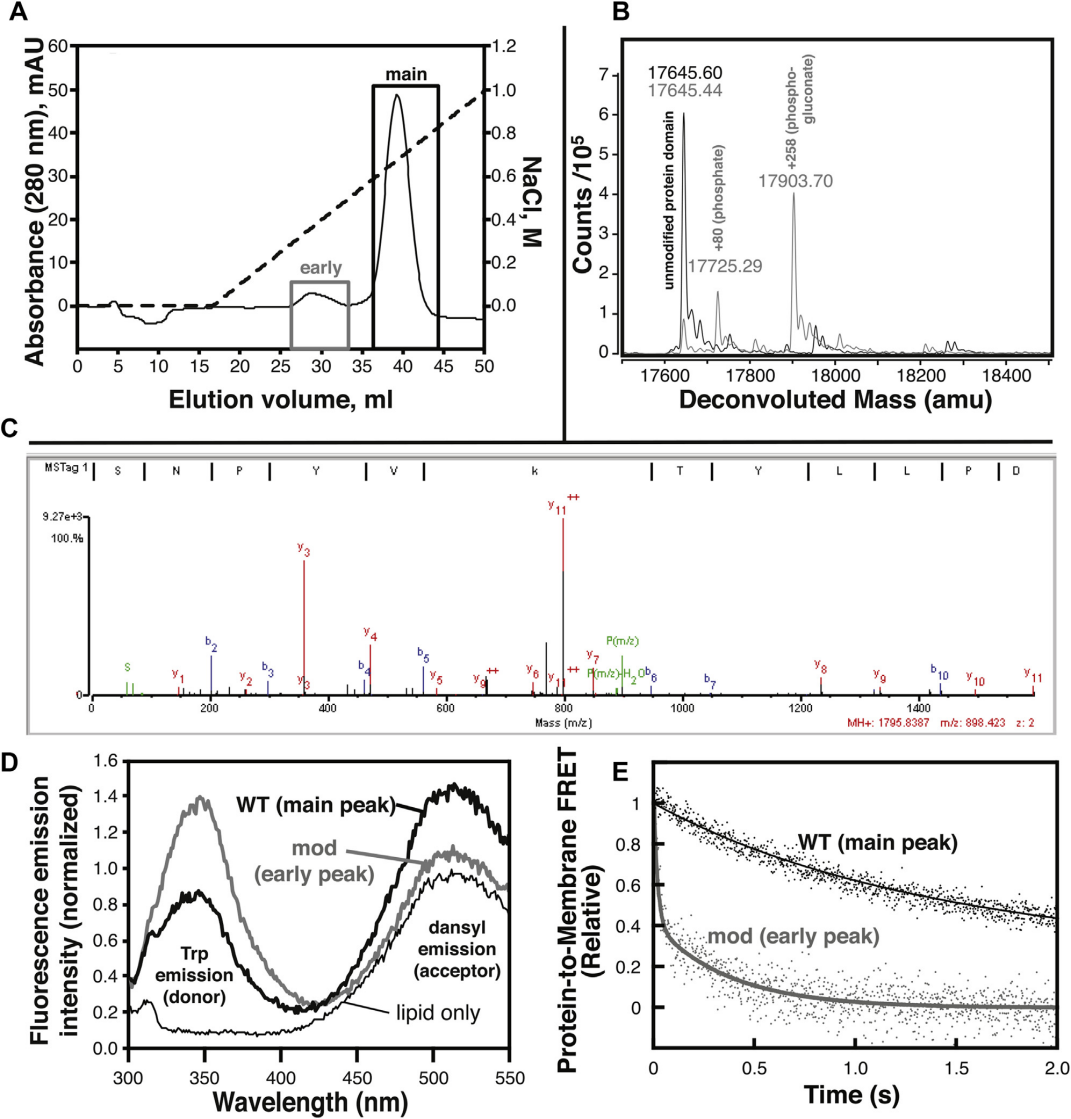
**Bacterial post-translational modifications weaken Slp-4 C2A membrane binding**. *A*, chromatogram from cation-exchange protein purification illustrating the two populations of protein collected. *Dashed line* shows the salt gradient used for elution. *B*, deconvoluted ESI-QTOF mass spectra of intact protein domains collected from the main (*black*) and early (*gray*) peaks in the chromatogram. *C*, annotated MS/MS fragmentation spectrum, including b and y ions, from tandem mass spectrometry of trypsin-digested protein from the early peak. The precursor ion corresponds to the peptide SNPYVkTYLLPD, where k represents phosphogluconoylated lysine at position K398. *D*, fluorescence emission spectra of PM liposomes before (*thin line*) and after (*thick lines*) addition of proteins from the early peak (*gray*) or main peak (*black*). The emission intensity increase at the dansyl (acceptor) peak is indicative of protein-membrane binding. *E*, kinetic measurement of dissociation from PM liposomes for the unmodified protein (*black*, same as Figure 3), and modified protein (*gray*). ESI-QTOF, electrospray ionization-quadrupole time-of-flight; PM, plasma membrane; Slp-4, synaptotagmin-like protein 4.

During protein purification, we noticed that a significant protein peak eluted early during cation-exchange chromatography (Fig. 9*A*). This is similar to a phenomenon that we and others have reported previously for cationic synaptotagmin C2 domains (24, 25, 26). We measured the precise molecular mass of the protein from this peak and found that the most abundant component had a mass greater than predicted (and compared to the protein in the main peak) by 258 Da (Fig. 9*B*). A 258-Da mass increase has been reported previously at the N-terminus of bacterially expressed proteins containing an N-terminal His tag, corresponding to a phosphogluconoyl modification arising from reaction of the terminal amino group on the protein with bacterial phosphogluconolactone (27, 28).

As our protein expression system lacks a His tag, we sought to identify the site(s) of modification by performing liquid chromatography with tandem mass spectrometry experiments on trypsin digests of samples from both peaks. Analysis using proteomics software confirmed the phosphogluconoyl modification on Lys398 in protein from the early peak, for which the fragment ion spectrum contains peaks for the full complement of y-ions as well as most of the b-ions (Fig. 9*C*). In addition, we noted another probable phosphogluconoylation site on Lys412 which we identified via chemical formula matching and manual analysis. Its MS/MS fragmentation spectrum includes nearly all predicted y-ions, although no b-ions (Fig. S9). Modification of Lys398 and Lys412 within the lysine cluster must be mutually exclusive because the whole-protein data only indicate a 258-Da mass increase (Fig. 9*B*). Thus, the lysine cluster of the Slp-4 C2A domain is susceptible to modification by reaction with endogenous phosphogluconolactone during bacterial protein expression.

The protein isolated from the early chromatography peak was found to retain a modest binding activity toward PM liposomes, producing a dansyl emission increase that was ∼20% of that of the WT protein domain at the same concentration (Fig. 9*D*). It is not clear whether this protein-to-membrane FRET arises from the weak binding of the phosphogluoconoylated protein or from another population, for example, the phosphorylated (+80 Da) population also visible in the whole-protein mass spectra (Fig. 9*B*). Owing to this relatively low signal, stopped-flow dissociation kinetic data of the modified protein were significantly noisier than those of WT. However, they could be fit to single- or double-exponential profiles that consistently contained a component with a rate constant of 1.7 ± 0.9 s^−1^ among three independent measurements (two of three measurements also contained a faster component of ∼50 s^−1^) (Fig. 9*E*). This rate is much faster than the major population of the unmodified protein (Table 3), consistent with weaker but measurable membrane binding by the modified protein domain. Thus, these data complement our mutational results and show that even a charge-reversal modification does not completely block reversible liposome binding by the Slp-4 C2A domain.

## Discussion

The results presented here show that Ca^2+^-independent binding of the Slp-4 C2A domain to cellular membranes and liposomes with physiological lipid composition is strong, driven primarily by electrostatics, and persists even when the PIP_2_ binding site is mutated. In particular, we report that (i) physiological levels of PIP_2_ and background anionic lipids (PS and PI) both contribute to a comparable extent toward the thermodynamic stability of the membrane bound state (Table 2); (ii) computer modeling and experimental mutational analysis support the lysine cluster as the primary PIP_2_ binding site, whereas basic residues throughout a broad surface contribute to nonspecific anionic lipid binding (Table 4); and (iii) the lysine cluster is susceptible to modification by carbonyl compounds such as phosphogluconolactone during bacterial protein expression (Fig. 9).

### Comparison with other lipid-binding domains

The Ca^2+^-independent nature of the Slp-4 C2A domain contrasts with other well-studied C2 domains, such as those from conventional PKC isoforms, synaptotagmin-1, and rabphilins (29, 30, 31), and arises because of the absence of key aspartate residues in the β2–β3 and β6–β7 loops (which are called CBL1 and CBL3 in Ca^2+^-sensitive C2 domains). Rather, both C2 domains of Slp-4 lack a full complement of Ca^2+^-coordinating Asp residues but contain a consensus motif associated with PIP_2_ binding, including the lysine cluster in the β3–β4 region (16, 32). C2 domains that share these sequence properties include other Slp family C2A domains, Slp-4 C2B, phosphatidylinositol 3-kinase C2A, RIM1 C2B, and synaptotagmin-4 C2A (16, 33). This consensus motif is not found in the Ca^2+^-independent C2 domains of the phosphatase and tensin homolog (PTEN) and novel PKC isoforms, which have type II topology (17, 34). The mechanism described here is also distinct from the Ca^2+^-independent type I topology C2 domain from kidney and brain expressed protein (KIBRA), which binds phosphoinositides using the opposite face of the C2 domain (35).

We and others have shown that Slp-4 C2A binds to multiple phosphoinositide species, among which PI(4,5)P_2_ is presumed to be the dominant target because of its ubiquity in the PM (12, 20). The 10-fold enhancement of PIP_2_ affinity in the presence of background anionic lipids is reminiscent of pleckstrin homology domains that bind phosphoinositides, except that Slp-4 C2A has a measurable affinity for these background anionic lipids even in the absence of PIP_2_ (21, 36, 37).

### Multiple lipid-binding sites

The biexponential dissociation kinetics observed in all of our samples (Table 3; Table S1) indicate that the protein exists in more than one lipid-binding state. The slowest off rate, ∼0.1 s^−1^, was observed only in lipid compositions containing both PIP_2_ and background anionic lipids; therefore, it most likely represents dissociation from a state with PIP_2_ bound in the lysine cluster and other anionic lipids associated with the broad cationic surface. Faster-dissociating states may reflect partial occupancy of the nonspecific surface and/or lipids other than PIP_2_ bound in the lysine cluster. The dissociation rate constant of these states tends to decrease (*i.e.*, tighter binding) as more PIP_2_ or PA is present, suggesting that these polyanionic lipids also bind in the nonspecific site(s) (Table 3; Table S1).

Binding of PIP_2_ lipids outside of the primary binding site was observed in two of our MD simulations. A PIP_2_ bound to the C-terminal lysine side chain in model 1, and two PIP_2_ molecules bound to the β2–β3 and β3–β4 loops, respectively, in model 2 (Table 4). Similarly, binding of phosphoinositide molecules outside of canonical binding sites has been reported computationally and experimentally for the PKCα C2 domain (38), as well as computationally for the general receptor for phosphoinositides (GRP-1) pleckstrin homology domain (39).

Although membrane binding is driven primarily by electrostatics, the Slp-4 C2A domain has one conserved hydrophobic residue that inserts into membranes, Phe452 on the β6–β7 loop. It is not clear why this insertion was captured in only one of three MD simulations of the WT protein (model 1, Table S4). The F452A mutation clearly decreased membrane binding in both MIN6 cells (Fig. 8) and our liposome binding assay (Fig. 6), indicating that its hydrophobic insertion also contributes to membrane binding by the Slp-4 C2A domain.

### Structural insights into membrane binding

Our simulation data indicate that nonspecific anionic lipid interactions include at least 13 basic residues spread over a broad surface of the protein domain (Table 4, Fig. 5*B*). These residues include all of the lysine and arginine residues on the lipid-facing surface of the protein except Arg454, which is located on the β6–β7 loop. Its α-carbon was among the closest to the membrane surface in all three PC/PS/PIP_2_ simulations (Table S4), and its side chain projects toward the membrane. Closer inspection revealed that the Arg454 side chain made extensive contact with the phosphodiester group of PC in all three simulations. Its lack of interaction with PS or PIP_2_ in these simulations could be incidental.

Consistent with a previous report, the K398A mutation effectively blocked binding to PIP_2_ (Fig. 6, red bars) (19). However, we show that this mutation leaves the protein's ability to bind background anionic lipids largely intact (Fig. 6, blue bars) and does not eliminate binding to cellular PMs (Fig. 8). In simulations of WT Slp-4 C2A, Lys398 had little interaction with PS, although it interacted extensively with PIP_2_ in model 3. Its position in the concave interior of the membrane-binding surface may make it more accessible to large phosphoinositide headgroups than to the smaller headgroups of PS or PA.

Another residue with interesting properties is Arg411. In the crystal structure, this side chain forms H-bonds with backbone oxygens on the β3–β4 loop and the β5 strand. A role of these H-bonds in stabilizing secondary structure could explain why the R411A mutant did not fold properly (Fig. S8). However, in our membrane-binding simulations of the WT protein domain, these H-bonds unraveled as the Arg411 side chain interacted with lipids. This loss of intraprotein H-bonding likely contributed to the increased conformational flexibility of the β3–β4 loop during membrane binding (Fig. S2). It is not yet clear how much this flexibility contributes to the membrane affinity of the domain.

### Significance of phosphogluconoyl modification

We observe that the major protein contaminant in the affinity-purified Slp-4 C2A domain contains a phosphogluconyl modification within the lysine cluster (Figs. 9 and S9). It has been shown previously that certain cationic, PIP_2_-binding C2 domains copurify with nucleic acids and other contaminants after bacterial expression and that these contaminants must be removed via ion-exchange chromatography to properly measure biophysical properties of the C2 domain (24, 25, 26). To our knowledge, this report is the first to identify and characterize a particular protein contaminant. We show that the modified protein binds membranes, albeit much more weakly than the unmodified protein (Fig. 9, *D* and *E*). This result underscores the importance of the ion-exchange step; because the contaminant retains some membrane binding activity, an incompletely purified protein would produce inaccurate results in binding and function assays.

The nature of the modification is surprising: phosphogluconoylation in bacterially purified proteins has been reported previously, but only at the amino terminus of an N-terminal His-tag (27, 28, 40). In contrast, our expression system used an N-terminal GST tag, which was cleaved before the cation-exchange step. The location of the modification suggests that the positive electrostatic environment (Fig. 1) imparts unusually low p*K*_a_ values to the lysine cluster side chains. This effect would make the amino groups more efficient nucleophiles for attacking reactive carbonyl compounds, the most abundant of which in *Escherichia coli* BL-21 strains happens to be phosphogluconolactone (27). We speculate that other C2 domains containing lysine clusters likely undergo the same modification.

The physiological significance of the modification is unclear. Although phosphogluconoylation is unlikely to be significant in eukaryotic cells, other carbonyl-containing compounds are known to react nonspecifically with lysines in various diseases, including diabetes and alcoholic liver disease (41, 42). Indeed, Slp-4 has been identified among the proteins modified by reactive lipid aldehydes in a mouse model of alcoholic fatty liver disease (43). Furthermore, lysines are the target of numerous enzymatic modifications, including acetylation and methylation, although it is unknown whether C2 domains are regulated this way. Based on the observation that mutations have a greater effect on membrane localization in MIN6 cells than on liposome binding under our *in vitro* assay conditions, we predict that lysine modification may significantly alter PM-binding properties of this protein. Further work is needed to clarify whether enzymatic or nonenzymatic modification of C2 domain lysine clusters plays a role in their function *in vivo*.

## Experimental and computational procedures

### Experimental materials and methods

#### Materials

Cholesterol, 1-palmitoyl-2-oleoyl-*sn*-glycero-3-phosphocholine (POPC, PC), 1-palmitoyl-2-oleoyl-*sn*-glycero-3-phosphoethanolamine, 1-palmitoyl-2-oleoyl-*sn*-glycero-3-phospho-l-serine, PI from the liver, PIP_2_ from the brain, and sphingomyelin from the brain were from Avanti Polar Lipids (Alabaster, AL). Dansyl-PE was from NOF America (White Plains, NY). IP_6_ dodecasodium salt was from Sigma. All reagents were of the American Chemical Society grade or higher.

#### Protein cloning, expression, and purification

Plasmid DNA encoding human Slp-4 (GenBank ID: BC014913.1) was obtained from American Type Culture Collection (Manassas, VA). The sequence encoding the C2A domain (residues G352-S494) was subcloned into a thrombin-cleavable glutathione-S-transferase fusion vector developed previously for bacterial protein expression and transformed into *E. coli* BL-21 DE3 cells (37, 44). Site-directed mutagenesis was performed using the QuikChange II XL kit (Agilent) following the manufacturer's instructions. All DNA sequences were verified using primer-extension sequencing (Eton Bioscience, San Diego, CA).

Bacterially expressed proteins were purified using glutathione affinity chromatography followed by cation exchange. Cells were lysed in the lysis buffer (50 mM Tris, 400 mM NaCl, 1% Triton X-100, 1 mM 2-mercaptoethanol (pH 7.5) with protease inhibitors) by sonication (Sonics Vibra Cell, Newtown, CT) using a 6-mm probe. Lysates were treated with DNAse I (2 U/ml) from Sigma (St Louis, MO) for 30 min, centrifuged to remove insoluble matter, and then supernatants were incubated with glutathione sepharose 4B beads (GE Healthcare, Chicago, IL) for 2 to 3 h at 4 °C. The beads were washed extensively with 50 mM Tris, 400 mM NaCl, 1 mM 2-mercaptoethanol (pH 7.5). and then with 50 mM Tris, 1.1 M NaCl, 5 mM EDTA, 1 mM 2-mercaptoethanol (pH 7.5). Beads were then exchanged into 50 mM Tris, 150 mM NaCl, 0.05 mM EDTA, 1 mM 2-mercaptoethanol (pH 7.7) for cleavage with restriction-grade thrombin (Millipore Sigma, Billerica, MA) and eluted using the thrombin cleavage buffer or buffer A (25 mM HEPES, 15 mM NaCl, 140 mM KCl, 0.5 mM MgCl_2_, pH 7.4) including 1 to 10 mM 2-mercaptoethanol. Cation-exchange chromatography was then performed using an Akta Purifier FPLC system with a HiTrap SP HP column (GE Healthcare), eluting with a gradient of NaCl. Eluted proteins were concentrated using Amicon centrifugal concentrators, purity was assessed using SDS-PAGE, and concentrations were measured based on absorbance at 280 nm using an extinction coefficient of 19,060 M^−1^ cm^−1^. Purified proteins were aliquoted, flash-frozen, and stored at −80 °C and were centrifuged after thawing for 2 min at 17,000*g* to remove any debris.

#### Liposome preparation

Phospholipids in chloroform were combined at the desired molar ratio for each experiment (Table 1). Lipid films were dried under vacuum for ≥2 h and rehydrated in buffer A. Small unilamellar vesicles were prepared by sonication to clarity using a Sonics Vibra Cell sonicator with a 3-mm tip. Liposomes were stored at 4 °C for at least 8 h after preparation before use and were used within 1 week. Lipid concentrations are reported as total accessible lipids, which is approximated as one-half of the total lipids.

#### Equilibrium protein-to-membrane FRET measurements

Equilibrium protein-to-lipid (Trp-dansyl) FRET titrations were performed as described previously (20). Measurements were made using a Photon Technology International QuantaMaster fluorescence spectrometer at 25 °C, with excitation at 284 nm (1-nm slit width) and emission at 520 nm (8-nm slit width). Protein (1 μM) was premixed with liposomes (125 μM total lipid) in buffer A, and fluorescence was measured upon titration with IP_6_ or NaCl. In a parallel sample, the titrant was added to solutions of lipid alone to correct for titrant effects on dansyl fluorescence. For the NaCl titrations, a second correction was made for signal loss upon the addition of the buffer to protein–lipid mixtures. Samples were equilibrated for 40 s with stirring after each addition. For the IP_6_ titrations, the resulting plot of intensity *F* vs. inhibitor concentration [IP_6_] was fit to a hyperbolic model for single-site competitive inhibition:

(1) $$F=\Delta F_{\text{max}}\left(1-\frac{\left[{\text{IP}}_{6}\right]}{{\text{IC}}_{50}+\left[{\text{IP}}_{6}\right]}\right)+C$$

where Δ*F*_max_ is the total FRET signal before IP_6_ titration, and IC_50_ is the IP_6_ concentration at which FRET is 50% of the initial value. (We note that the initial FRET in the absence of IP_6_ does not necessarily represent complete membrane binding.) To simplify graphical representations, data were normalized such that *C* = 0 and Δ*F*_max_ = 1.

To compare affinities among membranes of different target lipid compositions, it is both convenient and thermodynamically accurate to model the protein–membrane interaction as partitioning between an aqueous phase and a membrane phase, represented by a mole-fraction partition coefficient, which we denote *K*_x_ (45). This thermodynamic constant was calculated from the measured IC_50_ value as follows:

(2) $$K_{x}=\left(\frac{IC_{50}}{K_{I}}-1\right)\times \frac{\left[{\text{H}}_{2}\text{O}\right]}{\left[L\right]}$$

where *K*_I_ is the dissociation constant for protein-IP_6_ binding, reported previously to be 1.8 ± 0.1 μM (20), [H_2_O] is the bulk water concentration, and [*L*] is the total concentration of accessible lipids in the outer leaflet (*i.e.*, half the total bulk lipid concentration). Free energies of binding were then calculated as follows:

(3) $$\Delta G{}^{\circ}=-RT\;\ln\left(K_{x}\right)$$

where *R* is the gas constant and *T* is the temperature.

For comparison of liposome binding among different mutants, protein-to-membrane FRET measurements were performed using a Cytation 3 fluorescence plate reader (BioTek Instruments, Winooski, VT) with UV-transparent plates (Corning product no. 3679). Tryptophan-to-dansyl FRET was quantified by measuring tryptophan fluorescence emission in solutions containing the indicated mutant proteins (1 μM, concentrations checked via absorbance immediately prior) in buffer A with 1-mM tris-(2-carboxyethyl) phosphine, before and after the addition of liposomes (65 μM total accessible lipid). The percentage decrease in tryptophan emission was corrected for intrinsic Trp emission changes upon membrane binding (measured in separate wells using nonfluorescent liposomes) and then normalized to that of the WT protein domain.

#### Stopped-flow fluorescence spectroscopy

Stopped-flow fluorescence kinetic measurements were performed using a BioLogic SFM3000 spectrophotometer (Knoxville, TN) using 284-nm excitation and a 455-nm long-pass emission filter. To measure apparent on-rates (*k*_obs_), 1.2 ml of a solution containing protein (0.6 μM) was rapidly mixed with an equal volume of solution containing liposomes (75 μM total accessible lipid) in buffer A with 100 μM EDTA. Protein-to-membrane FRET (dansyl-PE emission) was monitored over time (*t*) for at least 8 replicate shots per sample, which were averaged and fitted to a single-exponential function (Equation 4):

(4) $$F=\Delta F_{\text{max}}\left(1-e^{-k_{\text{obs}}t}\right)+C$$

For off-rates (*k*_off_), protein-to-membrane FRET (dansyl-PE emission) was monitored after rapid mixing of equal volumes of protein-bound liposomes (75 μM total accessible lipid, 0.6 μM protein) and unlabeled liposomes in buffer A. Data sets were calculated as the average of 8 or more replicate shots per sample and were fitted to a single- or double-exponential function (Equation 5 or 6, respectively):

(5) $$\;F\;=\;\Delta F_{\text{max}}\left(e^{-k_{\text{off}}t}\right)\;+\;C\;$$

(6) $$\;F\;=\;\Delta F_{\text{max}1}\left(e^{-k_{\text{off}1}t}\right)\;+\;\Delta F_{\text{max}2}\left(e^{-k_{\text{off}2}t}\right)\;+\;C$$

where the *k*_off_ are dissociation rate constants and *C* are offsets.

For simplified presentation, *C* was subtracted and Δ*F*_max_ (or Δ*F*_max1_ + Δ*F*_max2_) was normalized to unity in the figures shown. Rate constants listed in Table 3 are the average ± SD of ≥3 independent samples. Dead time is estimated to be 1.4 ms.

The reported association rate constants *k*_on,x_ were calculated from the measured *k*_obs_ and *k*_off_ values using the mole-fraction partitioning model (45):

(7) $$k_{\text{on},\text{x}}=\left(k_{\text{obs}}-k_{\text{off}}\right)\times \frac{\left[{\text{H}}_{2}\text{O}\right]}{\left[L\right]}$$

#### Cloning and expression in MIN6 cells

Constructs for mammalian cell expression were generated from a plasmid encoding full-length rat Slp-4 in the pmCherry-C1 vector (gift from Ed Stuenkel, University of Michigan). (In this article, we use the numbering of the human protein for consistency; the rat numbering is the one greater because of one additional residue in the N-terminal region of the protein).

The plasmids encoding mCherry fused to the Slp-4 C2A domain (G352-A494) were prepared from the full-length mCherry–Slp-4 construct as follows: first, site-directed mutagenesis (QuikChange II XL, Agilent Technologies) was used to insert EcoRI restriction sites flanking the sequence encoding the N-terminal region of Slp-4 and to insert a stop codon 3ʹ to the C2A domain sequence; second, the sequence encoding the N-terminal region of Slp-4 was removed via EcoRI digestion and religation; third, the desired C2A domain mutations were introduced via site-directed mutagenesis. All DNA sequences were verified using primer-extension sequencing (Eton Bioscience, San Diego, CA).

MIN6 cells were cultured in the Dulbecco's modified Eagle's medium supplemented with 4.5 g/L d-glucose, 2.5 mM l-glutamine, 10% fetal bovine serum, 0.9% penicillin/streptomycin, and 5 ml 2-mercaptoethanol per liter of the media. Cells were seeded in 35-mm glass-bottom dishes coated with poly-d-lysine at ∼8 × 10^5^ cells per dish and were transfected with the desired plasmid (2 μg DNA/ml) using Lipofectamine-2000 following manufacturer protocols upon reaching 90 to 95% confluence. Cells were maintained in a 5% CO_2_ humidified atmosphere at 37 °C.

#### Fluorescence microscopy

Cells were imaged 60 h after transfection using a Zeiss Axio Observer fluorescence microscope with a 100× objective, with illumination from a 120-W lamp (Lumen Dynamics). The researchers were blinded to the sample identity during image acquisition and analysis.

#### Mass spectrometry: intact protein analysis

Purified bacterially expressed and cation exchange separated Slp-4 samples were desalted using C18 ZipTips (EMD Millipore). Desalted samples were diluted with 3% acetonitrile (ACN) in 0.1% formic acid to a final concentration of 100 ng/μl. Samples were chromatographically resolved on-line using a 2.1 × 50 mm, 5.0-μ PLRP-S 1000A column (Agilent Technologies) using a 1290 Infinity II LC system (Agilent Technologies). Mobile phases consisted of water + 0.1% formic acid (A) and 90% aq. ACN +0.1% formic acid (B). Samples were chromatographically separated using a flow rate of 0.3 ml/min using a gradient holding 5% B for 2 min and 5 to 95% B over 3 min for a total 5-min gradient. The gradient method was followed by a column wash at 95% B for 2 min before returning to the initial condition over 2 min. Data were collected on a 6550 Q-TOF equipped with a dual jet stream source (Agilent Technologies) operated in the MS-only mode. MS data were collected in positive-ion polarity over mass ranges 100 to 3200 m/z at a scan rate of 1.5 spectra/sec. Intact protein spectra were deconvoluted using maximum entropy in MassHunter Bioconfirm software (Agilent Technologies) to determine the accurate mass of unmodified and modified protein species if present.

#### Mass spectrometry: peptide analysis

Purified Slp-4 samples were tryptically digested using a protein digestion method previously described (46). Samples were reconstituted in 3% ACN + 0.1% formic acid and were chromatographically resolved on-line using a 2.1 × 250 mm, 2.7-μ AdvanceBio peptide mapping column (Agilent Technologies) using a 1290 Infinity II LC system (Agilent Technologies). Mobile phases consisted of water +0.1% formic acid (A) and 90% aq. ACN +0.1% formic acid (B). Samples were chromatographically separated using a flow rate of 0.2 ml/min using a gradient holding 5% B over 1 min, 5 to 40% B over 9 min, and 40 to 90% B over 2 min, for a total 12-min gradient. The gradient method was followed by a column wash at 90% B for 3 min before returning to initial conditions over 2 min. Data were collected on a 6550 Q-TOF equipped with a dual jet stream source (Agilent Technologies) operated using intensity-dependent collision-induced dissociation MS/MS to generate peptide IDs. MS/MS data were collected in positive-ion polarity over mass ranges 270 to 1700 m/z at a scan rate of 10 spectra/s for MS scans and mass ranges 50 to 1700 m/z at a scan rate of 3 spectra/s for MS/MS scans. All charge states were allowed, except singly charged species were excluded from being selected during MS/MS acquisition, and charge states 2 and 3 were given preference. SpectrumMill software (Agilent Technologies) was used to extract, search, and summarize peptide identity results. Spectra were searched against a custom database containing the Slp-4 protein amino acid sequence allowing up to 2 missed tryptic cleavages with fixed carbamidomethyl (C) and variable deamidated (NQ), oxidation (M), and phosphogluconoyl (K) modifications. The monoisotopic peptide mass tolerance allowed was ±20 ppm, and the MS/MS tolerance was ±50 ppm. A minimum peptide score of 8 and a scored peak intensity of 50% were used as cutoffs for identification of peptides.

### MD simulations and docking calculations

#### Stand-alone membrane model

Two different lipid bilayer membrane models were constructed using the CHARMM-GUI membrane builder (47), each with 256 lipids (128 per leaflet) in the *x-y* plane. The first model was made of pure POPC (100% PC), and the second model had mixed POPC:1-palmitoyl-2-oleoyl-*sn*-glycero-3-phospho-l-serine (190:64, a molar ratio of ∼3:1) with two molecules of 1-stearoyl-2-arachidonoyl-sn-glycero-3-phospho-(D)-myoinositol-(4,5)-bisphosphate (PIP_2_) placed on the protein-proximal leaflet for an effective 2% PIP_2_ density. The lipids were described by CHARMM36 force fields with updates for lipids and were hydrated with pre-equilibrated TIP3P (48, 49, 50) water models. Randomly selected water molecules were replaced with potassium (K^+^) and chloride (Cl^–^) ions to reach 0.15 M concentration and also for neutralizing the model system. Corrections recommended for the Lennard-Jones potential between K^+^ and lipid oxygens were included to avoid unnaturally strong binding of K^+^ to anionic lipids (51).

Each solvated model was minimized for 21,000 steps followed by equilibration for 2.8 ns at 1 bar and 298 K using nanoscale molecular dynamics (NAMD), version 2.10 (52). The production run of equilibration was then extended for 200 ns under the same conditions. The temperature was controlled with Langevin dynamics, where the temperature dampening coefficient was set to 1.0 and pressure controlled using the Langevin piston method (53, 54) with an oscillation period of 75 fs and a damping timescale of 25 fs. Long-range electrostatic interactions were computed using the particle mesh Ewald method (55, 56), and the short-range nonbonded interactions cutoff was set to 12 Å with a switching function set to 11 Å. The SHAKE algorithm was used to make waters rigid and to constrain all bonds between hydrogens and heavy atoms (57). A time step size of 2 fs was adopted. Unless otherwise stated, the same parameters were applied in the dynamics simulations throughout this study. The constructed membrane models were validated by examining the area per lipid (10.13039/501100000978APL) and order parameters (*S*_CH_) (58), which were found to be close to available literature values (51, 59, 60, 61) (see Additional details of methods in the Supporting information).

#### Stand-alone protein model

The crystal structure of the Slp-4 C2A domain (PDB: 3FDW) with all hydrogens added was solvated in the TIP3P water box at a physiological salt concentration (0.15 M KCl) and pH 7. The protein was described by the CHARMM36 force fields. The model was minimized for 10,000 steps with the protein backbone atoms (N, Cα, C, and O) frozen. The system was then slowly heated from 0 to 298 K for 40 ns with the backbone frozen. The model was further equilibrated for 2 ns at 298 K without constraints. Finally, an extended production run was performed for 200 ns. The trajectory was saved every 100 ps. Snapshots extracted from the saved trajectory were used in subsequent docking calculations and in the building of protein–membrane complex models. The electrostatic potential maps (+1.0 kT/e in blue and −1.0 kT/e in red, electrostatic equipotential contours) were calculated using APBS-PDB2PQR (62, 63) at pH 7.0 with 0.15 M KCl.

The protein exhibited certain conformational changes involving mostly the following regions: (i) A386 to K390 of the β2–β3 loop, (ii) H449 to N455 of the β6–β7 loop, (iii) P403 to G409 of the β3–β4 loop, and (iv) K398/K410/K412, which comprise the lysine cluster. To describe the conformation of the protein, we defined two angles using the tips of all three defined loops (β2–β3, β6–β7, and β3–β4) and the COM of the protein (Fig. S2*A*). The tip of each loop was defined as the COM of the C_α_ of three consecutive residues. More specifically, the tip of β2–β3 was represented by E388, A389, and K390; the tip of β6–β7 by G450, R451, and F452; and the tip of β3–β4 by S406, R407, and Q408. For example, in the crystal structure, the angle between β2–β3 and β6–β7 (which we term angle *α*) is 35°, and the adjacent angle between β6–β7 and β3–β4 (which we term angle *β*) is 76°.

#### Docking calculations

To determine the location of PIP_2_ binding to the surface of the Slp-4 C2A domain, docking calculations were performed with IP_3_ (the soluble analogue of the PIP_2_ head group) as the ligand and the entire C2A domain (either WT or mutant) as the receptor using the FlexiDock module in SYBYL 8.0. For the WT protein, we used the experimental protein structure (PDB: 3FDW) and three equilibrated protein structures extracted from the simulated trajectory of the stand-alone protein at *t* = 9.2, 120, and 200 ns, showing different protein conformations. *In silico* mutations were performed on the two equilibrated WT protein structures (*t* = 9.2 and 200 ns) using the SYBYL 8.0 mutation tool, followed by geometry minimizations. The ligand and proteins were described by the Tripos force fields (64). The Kollman (65) all-atom charges were assigned to the protein and Gasteiger-Huckel (66, 67) charges to the ligands. Rotatable bonds were allowed in the ligand but not in the protein. The docked poses were clustered (see Additional details of methods in the Supporting information), and the largest cluster can be interpreted as the most probable location of IP_3_ binding.

#### Protein–membrane complexes

We first built the complex model for the WT protein and membranes. Briefly, the last snapshot of the trajectory (*i.e.*, *t* = 200 ns) of the equilibrated stand-alone WT protein and membrane structures were merged using CHARMM (c40b1) and Visual Molecular Dynamics (VMD) (68, 69). We constructed four protein–membrane complex models: a PC control featured the POPC membrane model and models 1 to 3 the mixed PC/PS/PIP_2_ membrane model (Fig. S3). In all models, the protein was placed above the membrane with the protein COM ∼21 to 25 Å above the average PO_4_ plane of the upper leaflet of the lipid bilayer. In models 1 to 3, the proteins faced the membrane surface differently: (i) model 1 had the β2–β3 and β6–β7 loops positioned between the two PIP_2_ lipids such that each PIP_2_ lipid was of approximately equal distance from both loops, distances of 25 to 30 Å. (ii) Model 2 was made by rotating the protein in model 1 approximately 90° counterclockwise about the *z*-axis. The distance between one PIP_2_ and the loops region was about the same as the distance between the other PIP_2_ and the β4 binding site (∼15–20 Å). (iii) In model 3, β4 in the protein was positioned directly above one of the PIP_2_ lipids. Each model was minimized for 21,000 steps and equilibrated for 2.8 ns, followed by a production run for 200 ns using NAMD.

Next, we constructed the complex models for two single mutants K398A and F452A and two triple mutants R451A/F452A/R454A and K398A/R451A/R454A using the same protocol as for WT except for some minor differences. Here, we assume that the structures of these mutants and the WT domain are similar; this assumption is consistent with currently available experimental results and constraints. A mutant complex model used the mixed PC/PS/PIP_2_ membrane model and a starting protein geometry identical to model 3 of the WT protein. The selected residue(s) was(were) first mutated *in silico* and minimized while keeping the rest of the protein frozen. The numbers of K^+^ and Cl^–^ were adjusted to keep the system charge neutral and at 0.15 M salt concentration. The mutant complex model was then minimized for 10,000 steps and equilibrated for 5 ns, followed by production runs in the same way as for the WT complex models.

#### Depth penetration calculations

Depth penetration calculations were carried out to highlight penetration that occurred in the targeted regions. The position of the membrane was defined as the average *z*-coordinate of all the phosphorus atoms in the protein-facing leaflet of the bilayer. The selected residues were represented as the depth of their C_α_ atoms, except where noted. The membrane plane was set as the reference for the depth penetration calculated as the difference between the *z*-coordinates of the C_α_ of the given residue and of the membrane plane, with more negative values corresponding to deeper insertion.

#### Electrostatic contacts

Electrostatic contacts were defined as the selected basic residue side chains being within a cutoff distance from the heavy atoms of anionic lipid headgroups (from the phosphodiester linkage outward) in the model systems containing PS and PIP_2_. Calculations were performed for arginine and lysine as well as for histidine (H376, H476, and H481 as the HSE tautomer; H381, H448, and H449 as HSD), which was uncharged in all simulations. Cutoff distances were as follows: 5 Å for the amine nitrogen (N_z_) of lysine, 6.3 Å for the guadinino carbon (C_z_) of arginine, and 6.1 Å for the imidazole ring COM of histidine. For the PC control, similar calculations were performed for contacts with PC headgroups, considering heavy atoms from the glycerol backbone outward. Electrostatic lipid contacts were calculated independently for each residue; if a given residue was within the cutoff distance of one or more atoms of a particular lipid molecule, this was counted as a single contact.

## Data availability

The mass spectrometry data have been deposited to the ProteomeXchange Consortium via the PRIDE (70) partner repository with the data set identifier PXD021393. All other data are contained within the article.

## Conflict of interest

The authors declare that they have no conflicts of interest with the contents of this article.
