# Supplementary material for: Multivalent lipid targeting by the calcium-independent C2A domain of synaptotagmin-like protein 4/granuphilin
Source: J Biol Chem. 2020 Dec 10;296:100159. doi: 10.1074/jbc.RA120.014618 (PMC7857503; doi:10.1074/jbc.RA120.014618)
Supplement: Supplementary material [file mmc6.pdf]

Supporting Information for:

Multivalent lipid targeting by the calcium-independent C2A domain of synaptotagmin-like protein 4/granuphilin

Aml A Alnaas[1], Abena Watson-Siriboe[1], Sherleen Tran[1], Mikias Negussie,[1] Jack A. Henderson[1], J. Ryan Osterberg[1], Nara Lee Chon[1], Beckston M. Harrott[1], Julianna Oviedo,[1] Tatyana Lyakhova[1], Cole Michel[2], Nichole Reisdorph[2], Richard Reisdorph[2], Colin T. Shearn[3], Hai Lin*[1], and Jefferson D. Knight*[1]

From the [1]Department of Chemistry, University of Colorado Denver, Denver CO 80217; [2]Department of Pharmaceutical Sciences and [3]Department of Pediatrics, Division of Pediatric Gastroenterology, Hepatology and Nutrition, School of Medicine, University of Colorado Anschutz Medical Campus, Aurora CO 80045.

*corresponding authors:

Hai.Lin@ucdenver.edu

Jefferson.Knight@ucdenver.edu

Table of Contents

Additional Details of Methods 3
Figure S1 4
Table S1 5
Figure S2 6
Table S2 7
Figure S3 8
Table S3 9
Figure S4 10
Figure S5 11
Table S4 12
Figure S6 13
Figure S7 14
Figure S8 15
Table S5 16
Figure S9 17
References for Supporting Information 18

Additional Details of Methods

Assessment of Membrane Models

To assess the constructed membrane models, area per lipid (APL) and order parameters (S_{CH}) were calculated. The APL was calculated by dividing the total area of lipid by the total number of lipids of each leaflet (128 lipids); the total area was averaged over the 200 ns of the productive run. The APL for pure POPC, 0.64 nm^2, matches closely to the reference APL 0.639 nm^2. (1,2) The S_{CH} describes the orientation of the lipid alkyl chains. The value of S_{CH} is determined by measuring the angle θ between the vector normal to the membrane surface and the vector of C-H bond of the lipid (Eq. S1). (3)

$$S_{\mathrm{CH}} = \frac{1}{2}(3\langle\cos^2\theta\rangle - 1) \tag{S1}$$

The brackets indicate the average over the two C-H bonds in a CH_2 group at a given position along the alkyl chain over all lipids of the same type and over the 200 ns of simulation time. The S_{CH} has yet to be determined for the arachidonic and stearic acyl chains of PIP_2. However, reference values for the oleoyl and palmitoyl acyl chain have been determined and are in close agreement with S_{CH} values found in this work. (4,5) We therefore conclude that the membrane models are valid for use in the subsequent simulations.

Docking Calculations and Clustering

For each protein conformation tested, 510 different initial positions of the ligand were distributed uniformly around the protein. The 510 docked poses were clustered based on the distances between the centers of mass of the ligands, using a cutoff distance of 10 Å. More specifically, we began with the pose of the highest fitness, which was the delegate of the first cluster. We then assigned to the first cluster the docked ligands whose centers of mass were within 10 Å of the center of mass of the first-cluster delegate. All poses in the first cluster were deleted from the list of 510 poses. Next, we identified the pose of the highest fitness among the remaining poses, made it the delegate of the second cluster, and included poses within 10 Å of the center of mass of this delegate into the second cluster. This process was repeated until every pose was assigned to a cluster. In this way, the clusters are mutually exclusive, because every pose can only be assigned to one cluster.

Figure S1

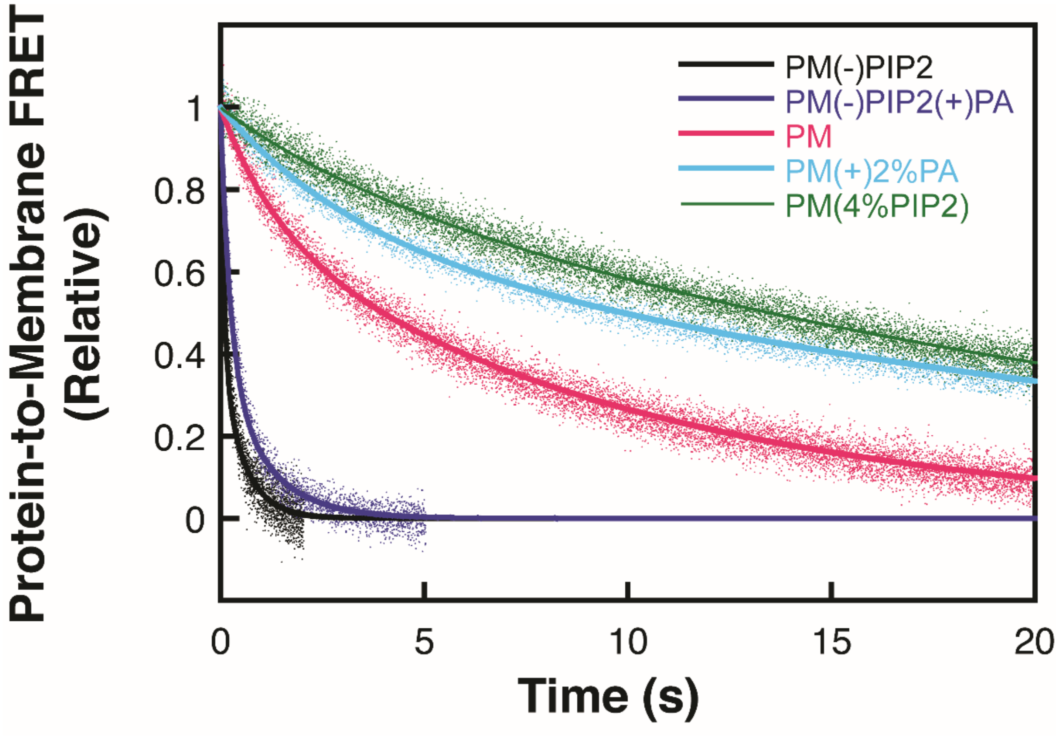

Figure S1: Effect of phosphatidic acid (PA) on wild-type Slp-4 C2A membrane dissociation kinetics. Kinetics were measured using stopped-flow fluorescence spectroscopy and fit as described in the Methods of the main text. Fit parameters are given in **Table S1**. PA has a small headgroup with a net charge of approximately -1.5 at pH 7.4. (6) Inclusion of 2% POPA in the liposome lipid composition decreased the off-rate by approximately a factor of two, both in the absence (compare dark blue to black curves) and in the presence (compare cyan to magenta curves) of 2% PIP_2. For comparison, inclusion of 2% PIP_2 decreased the off-rate by a factor of approximately 40 (compare magenta to black curves), but a further increase to 4% PIP_2 only decreased the off-rate further by a factor of approximately two (compare green to magenta curves). These results suggest that despite its polyanionic character, PA cannot substitute for PIP_2 in the PIP-selective binding site of the C2 domain, but rather slows protein dissociation through nonspecific secondary electrostatic interactions. The results are consistent with PIP_2 binding the protein both selectively (dominant at lower PIP_2 percentages) and through nonspecific electrostatic interactions (apparent at higher PIP_2 percentages).

Lipid compositions are based on those shown in **Table 1** of the main text; PA was substituted for PC, whereas PIP_2 was substituted for PI.

Table S1: Comparison of rate constants (k_1, k_2), amplitudes (Amp_1, Amp_2), and time to 50% dissociation ($t_{1/2}$) from the data shown in Figure S1. Two independent sets of measurements were performed and both values are shown here.

Lipid composition	Lipid comp alt	k_1 (s^{-1})	Amp_1 (%)	k_2 (s^{-1})	Amp_2 (%)	$t_{1/2}$ (s)
PM(-)PIP2	0% PIP2 0% PA	12, 10	60, 100	1.8, ND	40, 0	0.11, 0.07
PM(-)PIP2(+)PA	0% PIP2 2% PA	4.3, 8.0	62, 64	0.94, 1.0	38, 36	0.26, 0.17
PM	2% PIP2 0% PA	0.72, 1.35	28, 22	0.10, 0.17	72, 72	4.0, 2.7
PM(+)2%PA	2% PIP2 2% PA	0.31, 0.70	30, 35	0.037, 0.079	70, 65	9.9, 3.9
PM(4%PIP2)	4% PIP2 0% PA	0.30, 0.42	12, 20	0.042, 0.053	88, 80	13.6, 9.0

Figure S2

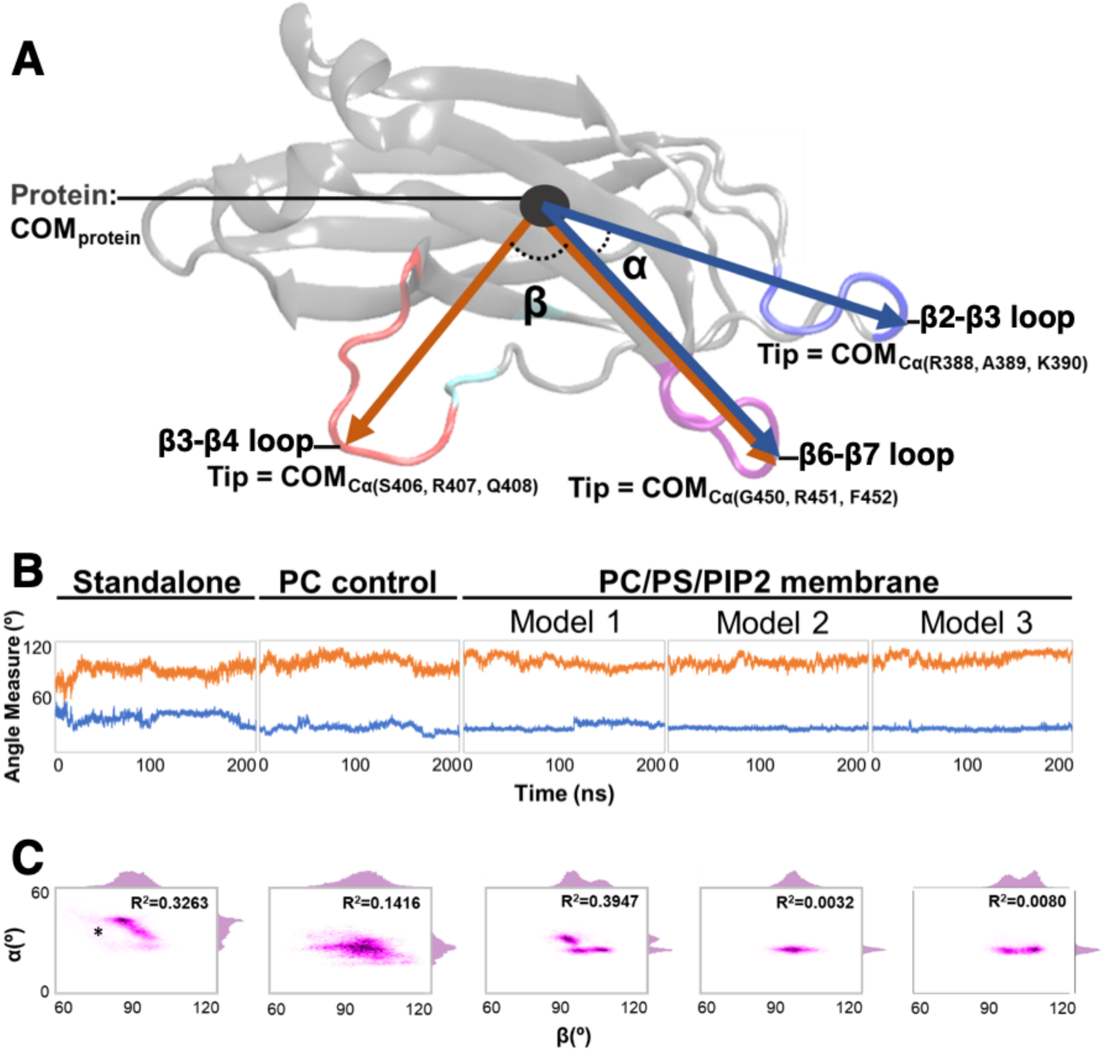

Figure S2: Protein conformational changes during MD simulations. **A:** Structure of the Slp-4 C2A domain illustrating the definitions of angles α and β which we use to report protein conformational changes. **B:** Angle values of α (blue) and β (orange) during MD simulations of the protein alone (left), or initially positioned above a lipid bilayer. **C:** Correlation plots of α and β during each simulation. Asterisk indicates α (35°) and β (76°) values in the crystal structure.

During the course of the standalone protein simulation, α decreased while β increased in a correlated manner, indicating movement of the β6-β7 loop toward the β2-β3 loop. The final conformation from the standalone simulation was used as the starting protein conformation in each of the simulations with lipids. Although α and β exhibited broad, uncorrelated dynamics in the PC control simulation, the simulations in the presence of anionic membranes confined α to a narrow range of values with the β6-β7 and β2-β3 loops in close proximity. Correlation analysis suggests the presence of up to three distinct conformational substates in these simulations. The greater variability of β reflects dynamics of the β3-β4 loop.

Table S2: Results of IP_3 docking calculations toward mutant forms of Slp-4 C2A. Docking calculations were performed using FlexiDock as described in the main text. The lysine cluster and loops cluster are defined as in **Figure 4A** of the main text. Mutations were generated computationally using the indicated starting structure from the standalone simulation of the wild-type protein domain.

Protein Structure	Snapshot 1 (9.2 ns) (α = 55°)			Snapshot 3 (200 ns) (α = 25°)		
	Lys cluster (%)	Loops (%)	Other (%)	Lys cluster (%)	Loops (%)	Other (%)
**Wild Type**	81	5	14	33	32	35
**K398A**	65	10	25	12	42	45
**K410A**	68	7	25	12	39	48
**K412A**	69	12	18	8	48	45
**K398A/K410A**	53	13	29	4	42	54
**K398A/K412A**	46	21	33	0	44	57
**K410A/K412A**	33	27	40	0	47	54
**K398A/K410A/K412A**	9	45	47	0	45	55

Figure S3

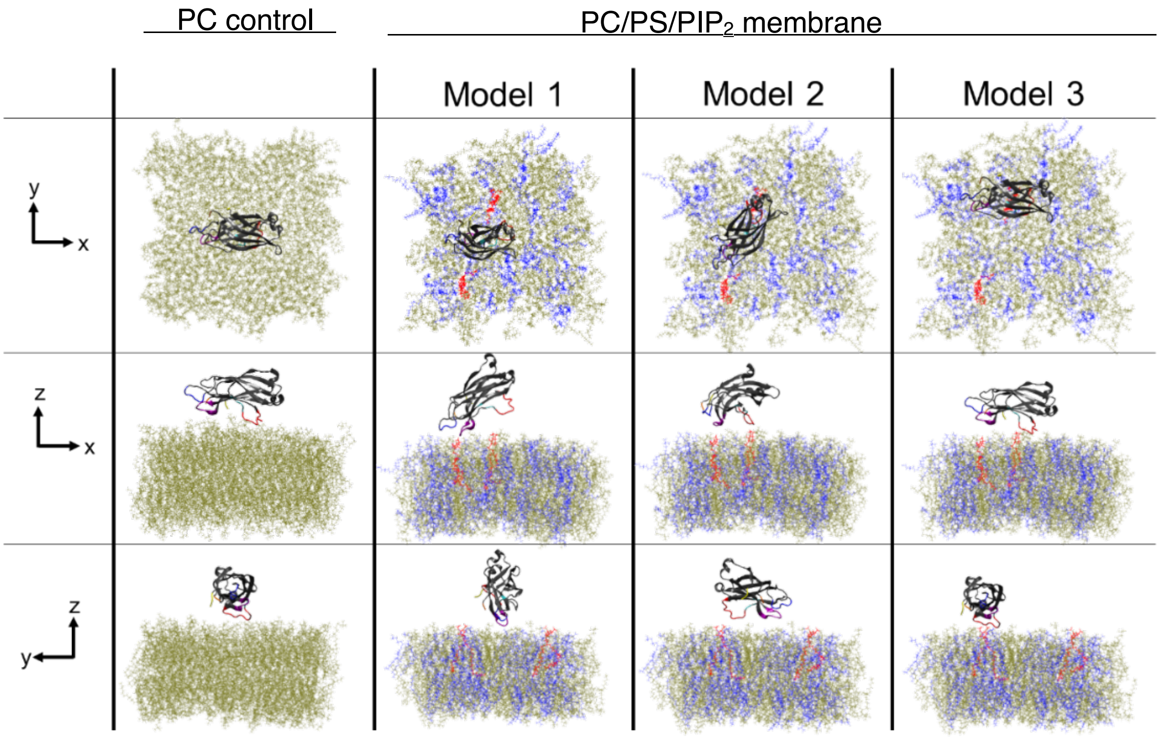

Figure S3: Initial geometries used for MD simulations with membranes. Each model comprises a protein membrane system (solvated in water) that was run for 200 ns. All models were solvated in 0.15 M KCl to maintain a physiological salt concentration. The initial protein conformation used for all simulations is the endpoint (200 ns) of the protein standalone simulation. In Models 1, 2, and 3 the protein domain (gray) sits on top of a membrane bilayer that is made up of 190 PC molecules (tan), 64 PS molecules (blue), and 2 PIP_2 molecules (red). These models differ in the initial placement of the protein domain and lipids. In Model 1, the protein domain was placed between the two PIP_2 molecules with its lysine cluster toward the membrane and its long axis approximately perpendicular to a line joining the PIP_2 molecules. In Model 2, the long axis was approximately parallel to a line joining the PIP_2 molecules; i.e., the two PIP_2 molecules were underneath the β2-β3 and β3-β4 loops, respectively. In Model 3, the protein was placed with its lysine cluster directly above one of the PIP_2 molecules. In the PC control, the protein was placed centrally with its initial orientation the same as Model 3.

Table S3: Full list of electrostatic contacts between basic residues and anionic lipids in the PC/PS/PIP_2 simulations. All basic residues are included. The number of lipid molecules contacted by each residue was averaged over the last 100 ns of simulations (± S.D.). Contacts were calculated as described in Methods. Secondary structural elements are listed on the left, and the conserved lysine cluster is highlighted blue.

		Model 1		Model 2		Model 3	
		Avg PS Contact	Avg PIP_2 Contact	Avg PS Contact	Avg PIP_2 Contact	Avg PS Contact	Avg PIP_2 Contact
β1	**R359**	0.00 ± 0.00	0.00 ± 0.00	0.00 ± 0.00	0.00 ± 0.00	0.00 ± 0.00	0.00 ± 0.00
	**K365**	0.00 ± 0.00	0.00 ± 0.00	0.01 ± 0.09	0.00 ± 0.00	0.00 ± 0.00	0.00 ± 0.00
β2	**H376**	0.00 ± 0.00	0.00 ± 0.00	0.00 ± 0.00	0.00 ± 0.00	0.00 ± 0.00	0.00 ± 0.00
	**K378**	0.00 ± 0.00	0.00 ± 0.00	0.00 ± 0.00	0.00 ± 0.00	0.00 ± 0.00	0.00 ± 0.00
	**H381**	0.00 ± 0.00	0.00 ± 0.00	0.00 ± 0.00	0.00 ± 0.00	0.00 ± 0.00	0.00 ± 0.00
β2- β3	**K390**	1.30 ± 0.79	0.00 ± 0.00	0.30 ± 0.45	0.10 ± 0.04	0.01 ± 0.11	0.00 ± 0.00
	**K391**	0.00 ± 0.01	0.00 ± 0.00	0.00 ± 0.00	0.00 ± 0.00	0.00 ± 0.00	0.00 ± 0.00
	**R392**	1.12 ± 0.01	0.00 ± 0.00	0.13 ± 0.34	1.00 ± 0.00	0.45 ± 0.69	0.00 ± 0.00
β3	**K398**	0.00 ± 0.04	0.00 ± 0.00	0.00 ± 0.00	0.00 ± 0.00	0.59 ± 0.49	1.00 ± 0.06
β3-β4	**K405**	0.00 ± 0.05	0.00 ± 0.00	0.01 ± 0.11	0.99 ± 0.10	0.33 ± 0.48	0.00 ± 0.00
	**R407**	0.08 ± 0.27	0.00 ± 0.00	0.19 ± 0.39	1.00 ± 0.00	0.90 ± 0.45	0.00 ± 0.00
	**K410**	0.00 ± 0.00	0.00 ± 0.00	0.00 ± 0.00	0.00 ± 0.00	0.37 ± 0.50	0.00 ± 0.00
β4	**R411**	1.59 ± 0.60	0.00 ± 0.00	0.19 ± 0.39	0.00 ± 0.00	0.65 ± 0.48	0.00 ± 0.00
	**K412**	0.78 ± 0.54	0.00 ± 0.00	0.12 ± 0.33	0.00 ± 0.00	0.02 ± 0.13	1.00 ± 0.00
β4-β5	**K416**	0.29 ± 0.51	0.00 ± 0.00	0.52 ± 0.52	0.00 ± 0.00	0.00 ± 0.04	0.00 ± 0.00
	**R417**	0.24 ± 0.45	0.02 ± 0.13	0.97 ± 0.55	0.10 ± 0.31	0.26 ± 0.49	0.01 ± 0.08
β5	**R429**	0.09 ± 0.29	0.00 ± 0.00	0.26 ± 0.49	0.00 ± 0.00	0.01 ± 0.10	0.00 ± 0.00
β6	**R440**	0.00 ± 0.00	0.00 ± 0.00	0.00 ± 0.00	0.00 ± 0.00	0.00 ± 0.04	0.00 ± 0.00
	**H448**	0.00 ± 0.00	0.00 ± 0.00	0.00 ± 0.00	0.00 ± 0.00	0.00 ± 0.00	0.00 ± 0.00
β6-β7	**H449**	0.02 ± 0.13	0.00 ± 0.00	0.17 ± 0.39	0.00 ± 0.00	0.01 ± 0.07	0.91 ± 0.29
	**R451**	0.00 ± 0.00	0.00 ± 0.00	0.52 ± 0.50	0.00 ± 0.06	0.51 ± 0.80	0.00 ± 0.00
	**R454**	0.00 ± 0.00	0.00 ± 0.00	0.00 ± 0.00	0.00 ± 0.00	0.12 ± 0.32	0.12 ± 0.33
β7-β8	**K469**	0.00 ± 0.00	0.00 ± 0.00	0.00 ± 0.00	0.00 ± 0.00	0.00 ± 0.00	0.00 ± 0.00
	**K472**	0.00 ± 0.00	0.00 ± 0.00	0.00 ± 0.00	0.00 ± 0.00	0.00 ± 0.00	0.00 ± 0.00
	**K473**	0.00 ± 0.00	0.00 ± 0.00	0.00 ± 0.00	0.00 ± 0.00	0.00 ± 0.00	0.00 ± 0.00
β8	**H476**	0.00 ± 0.00	0.00 ± 0.00	0.00 ± 0.00	0.00 ± 0.00	0.00 ± 0.00	0.00 ± 0.00
C-ter	**H481**	0.00 ± 0.00	0.00 ± 0.00	0.02 ± 0.15	0.00 ± 0.00	0.00 ± 0.00	0.00 ± 0.00
	**K483**	0.00 ± 0.00	0.95 ± 0.21	0.00 ± 0.00	0.00 ± 0.00	0.00 ± 0.04	0.00 ± 0.00

Figure S4

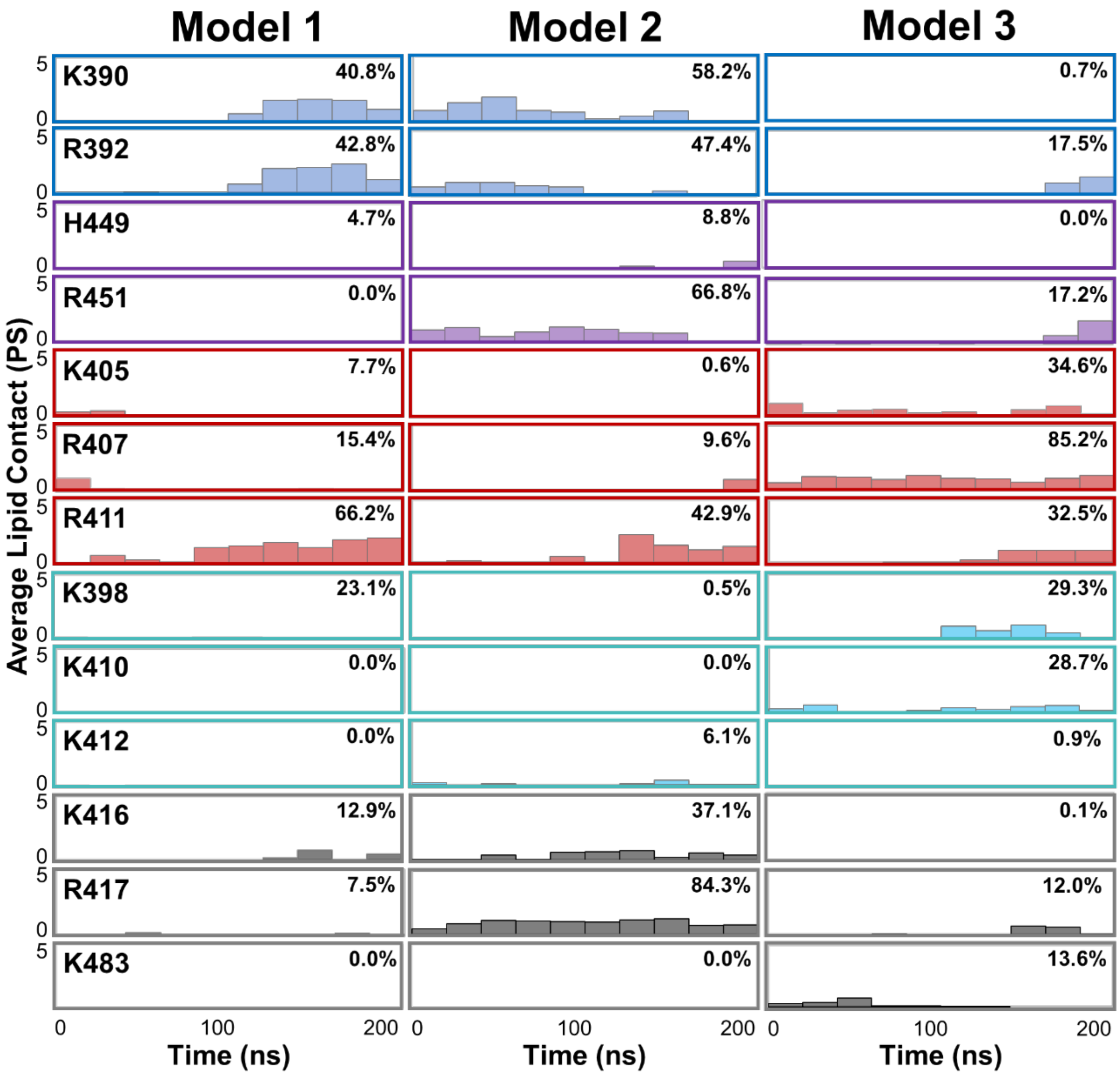

Figure S4. PS electrostatic contacts. Bars represent the POPS contact number (averaged over 20-ns intervals) for the thirteen residues with the most total electrostatic contact throughout the 200-ns simulations. The percentage in the top right of each graph represents the proportion of the simulation during which at least one contact was observed. Data sets are colored by structural element: β2-β3 loop (blue), β6-β7 loop (purple), β3-β4 loop (red), the conserved lysine cluster (cyan), and other residues in other regions of the protein (gray). Interactions with the β2-β3 loop were lowest in Model 3, while interactions with the β6-β7 and β3-β4 loops varied throughout the models. PS binding in addition to PIP_2 was detected in the lysine cluster site in Model 3. Arg411 interaction with PS increased over time in all of the simulations.

Figure S5

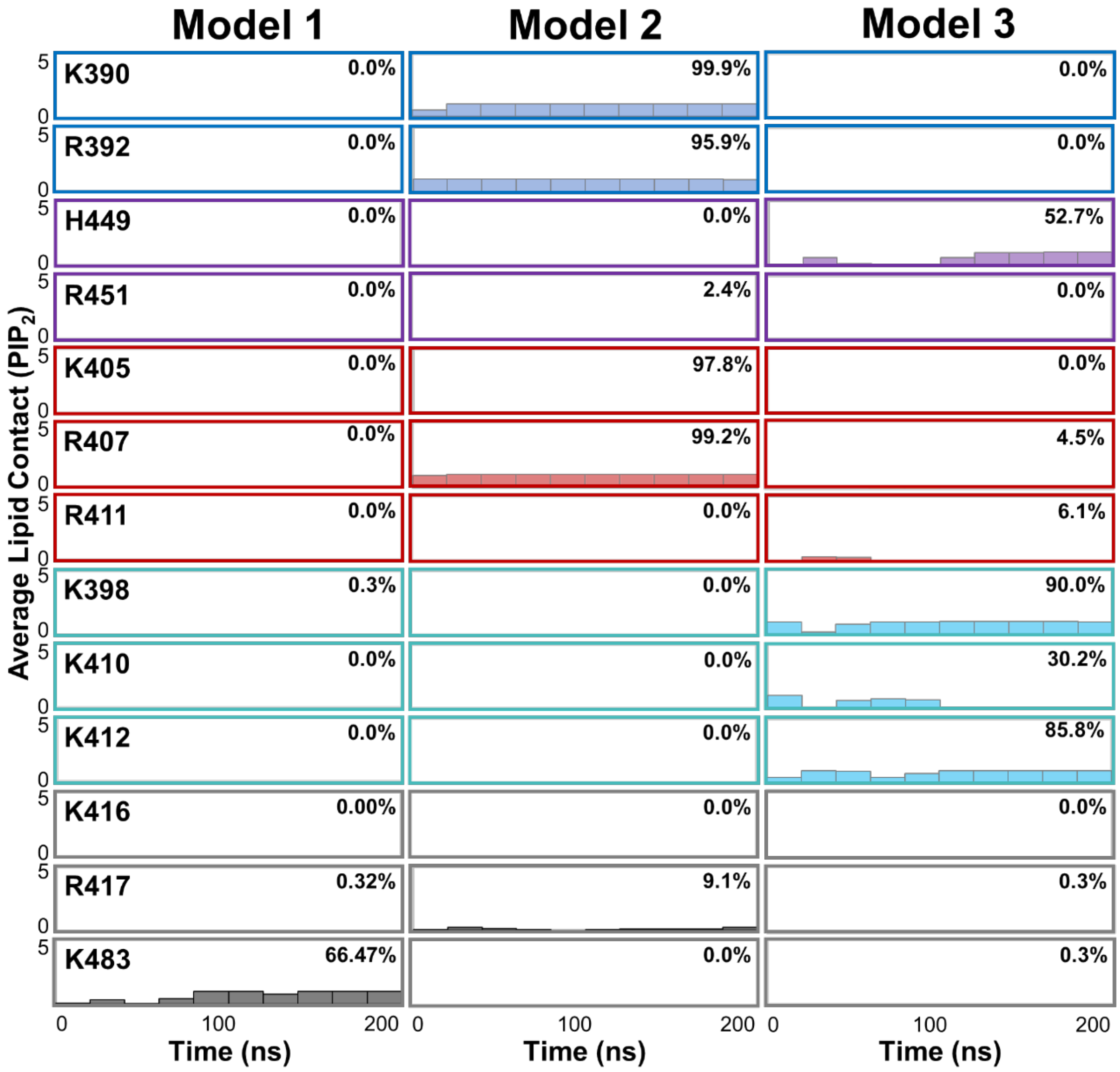

Figure S5. PIP_2 electrostatic hydrophilic contacts. Bars represent the PIP_2 contact number (averaged over 20-ns intervals) for the thirteen residues with the most total electrostatic contact throughout the 200-ns simulations. The percentage in the top right of each graph represents the proportion of the simulation during which at least one contact with PIP_2 was observed. Data sets are colored by structural element: β2-β3 loop (blue), β6-β7 loop (purple), β3-β4 loop (red), the conserved lysine cluster (cyan), and residues in other regions of the protein (gray). In all three models, PIP_2 interacted with the residues directly above it in the initial placement: K483 (C-terminus) in Model 1, the β2-β3 and β3-β4 loops in Model 2, and the lysine cluster plus H449 in Model 3. The contacts in Model 3 most closely resemble the previously reported binding surface for this domain (7) as well as other PIP_2-binding C2 domains. (8-10)

Table S4: Penetration depth (Å) of selected residues during MD simulations. Distances relative to the average phosphate plane of the membrane are presented for the given residues in the PC control and Models 1, 2, and 3. Data are presented as mean ± standard deviation (minimum/maximum) over the 200-ns simulation. All values shown are from backbone α-carbons, unless otherwise noted. Overall, most of the protein-lipid interactions are with the polar headgroup region (positive values) rather than below the phosphate plane (negative values).

		PC control	PC/PS/PIP2 membrane		
			Model 1	Model 2	Model 3
Protein		26.0 ± 2.4 (21.1 / 34.7)	14.7 ± 2.4 (10.3 / 20.9)	15.9 ±1.2 (12.8 / 20.5)	18.1 ± 2.5 (12.7 / 24.5)
β2-β3 loop	A386	28.2 ± 4.6 (15.5 / 39.0)	8.7 ± 1.5 (3.2 / 13.3)	12.6 ± 2.3 (6.7 / 19.3)	12.4 ± 3.2 (4.7 / 23.2)
	D387	28.0 ± 4.2 (17.7 / 39.6)	7.6 ± 1.7 (1.3 / 12.4)	10.9 ± 2.2 (5.2 / 17.9)	11.9 ± 3.7 (4.1 / 24.5)
	E388	30.0 ± 4.8 (19.4 / 42.9)	10.4 ± 2.0 (2.1 / 15.7)	11.3 ± 2.4 (7.5 / 20.2)	14.1 ± 3.8 (5.8 / 27.5)
	A389	32.7 ± 5.1 (21.2 / 46.0)	8.8 ± 2.1 (0.3 / 14.8)	11.3 ± 2.4 (5.3 / 19.2)	13.6 ± 4.2 (4.8 / 28.6)
	K390	32.8 ± 5.7 (18.7 / 45.9)	7.7 ± 1.7 (2.3 / 13.4)	11.8 ± 2.0 (5.6 / 19.0)	16.2 ± 4.9 (7.0 / 31.6)
β3-β4 loop	Y400	19.8 ± 2.4 (15.1 27.8)	14.7 ± 2.7 (6.4 / 18.2)	13.9 ± 1.2 (10.4 / 18.3)	13.7 ± 2.3 (8.4 / 21.4)
	L401	19.7 ± 2.9 (11.0 / 26.6)	13.5 ± 2.8 (7.8 / 20.2)	15.8 ± 1.4 (12.4 / 20.6)	19.7 ± 2.9 (11.0 / 26.6)
	L402	17.3 ± 3.1 (10.8 / 24.1)	13.6 ± 2.8 (9.9 / 20.1)	17.0 ± 1.3 (12.5 / 21.5)	13.7 ± 2.6 (7.1 / 22.2)
	P403	15.0 ± 3.3 (8.1 / 21.6)	12.6 ± 2.7 (6.7 / 19.4)	16.9 ± 1.5 (11.5 / 21.9)	12.1 ± 3.0 (4.6 / 22.1)
	D404	13.3 ± 2.7 (7.4 / 20.1)	9.4 ± 2.8 (3.6 / 16.4)	13.2 ± 1.5 (8.6 / 18.0)	9.7 ± 2.7 (3.2 / 18.9)
	K405	11.0 ± 2.1 (6.0 / 18.0)	7.7 ± 3.1 (1.4 / 15.5)	10.5 ± 1.6 (5.3 / 15.5)	8.6 ± 2.6 (2.6 / 18.6)
	S406	10.3 ± 1.9 (5.6 / 20.6)	5.7 ± 3.2 (-0.4 / 13.2)	7.8 ± 1.5 (3.9 / 12.5)	8.2 ± 2.4 (2.6 / 16.4)
	R407	9.6 ± 1.9 (4.5 / 17.2)	2.1 ± 3.4 (-4.4 / 10.7)	4.6 ± 1.5 (0.4 / 9.9)	5.3 ± 2.4 (-0.2 / 12.9)
	Q408	10.9 ± 2.2 (5.5 / 20.6)	1.7 ± 3.3 (-4.8 / 9.7)	4.1 ± 1.4 (0.3 / 9.4)	6.5 ± 2.3 (1.4 / 15.3)
	Q408[a]	8.4 ± 2.6 (2.7 / 21.8)	0.2 ± 4.2 (-8.3 / 12.0)	2.5 ± 1.8 (-2.6 / 11.1)	7.9 ± 2.6 (0.8 / 16.9)
	G409	14.1 ± 2.3 (8.3 / 23.0)	2.2 ± 2.9 (-3.8 / 9.5)	4.4 ± 1.3 (0.5 / 9.8)	6.4 ± 2.6 (1.0 / 14.7)
	K410	16.5 ± 2.4 (11.8 / 25.3)	5.6 ± 2.7 (-0.4 / 12.7)	7.6 ± 1.3 (3.9 / 12.9)	9.0 ± 2.3 (3.8 / 16.5)
β6-β7 loop	H449	23.3 ± 3.2 (15.5 / 39.0)	3.6 ± 1.8 (-0.3 / 9.4)	6.0 ± 1.5 (2.1 / 11.4)	8.7 ± 4.0 (1.4 / 19.3)
	G450	24.2 ± 3.7 (15.8 / 37.2)	2.3 ± 1.9 (-2.1 / 8.4)	5.8 ± 1.6 (1.4 / 11.3)	9.1 ± 4.4 (1.4 / 21.4)
	R451	22.7 ± 4.4 (13.4 / 37.8)	-1.0 ± 1.8 (-5.5 / 5.1)	3.8 ± 2.0 (-1.1 / 11.2)	6.6 ± 4.7 (-1.2 / 20.3)
	F452	20.8 ± 4.5 (11.3 / 34.7)	0.2 ± 1.6 (-4.4 / 5.5)	6.4 ± 2.5 (1.1 / 14.8)	6.6 ± 4.1 (-1.1 / 19.4)
	F452[b]	21.6 ± 5.5 (10.2 / 37.2)	-2.5 ± 1.9 (-7.7 / 4.0)	6.1 ± 2.3 (-0.9 / 14.9)	6.1 ± 4.1 (-2.8 / 19.9)
	G453	19.6 ± 4.7 (10.3 / 33.6)	2.9 ± 1.4 (-1.7 / 7.2)	8.2 ± 2.6 (2.1 / 16.6)	7.2 ± 3.5 (0.3 / 18.6)
	R454	18.1 ± 4.4 (9.3 / 30.7)	1.6 ± 1.5 (-1.8 / 6.3)	5.7 ± 2.3 (0.5 / 13.4)	5.8 ± 3.4 (-0.9 / 16.0)

[a]Reference point taken to be Cδ.

[b]Reference point taken to be the center of mass of the Phe ring.

Figure S6

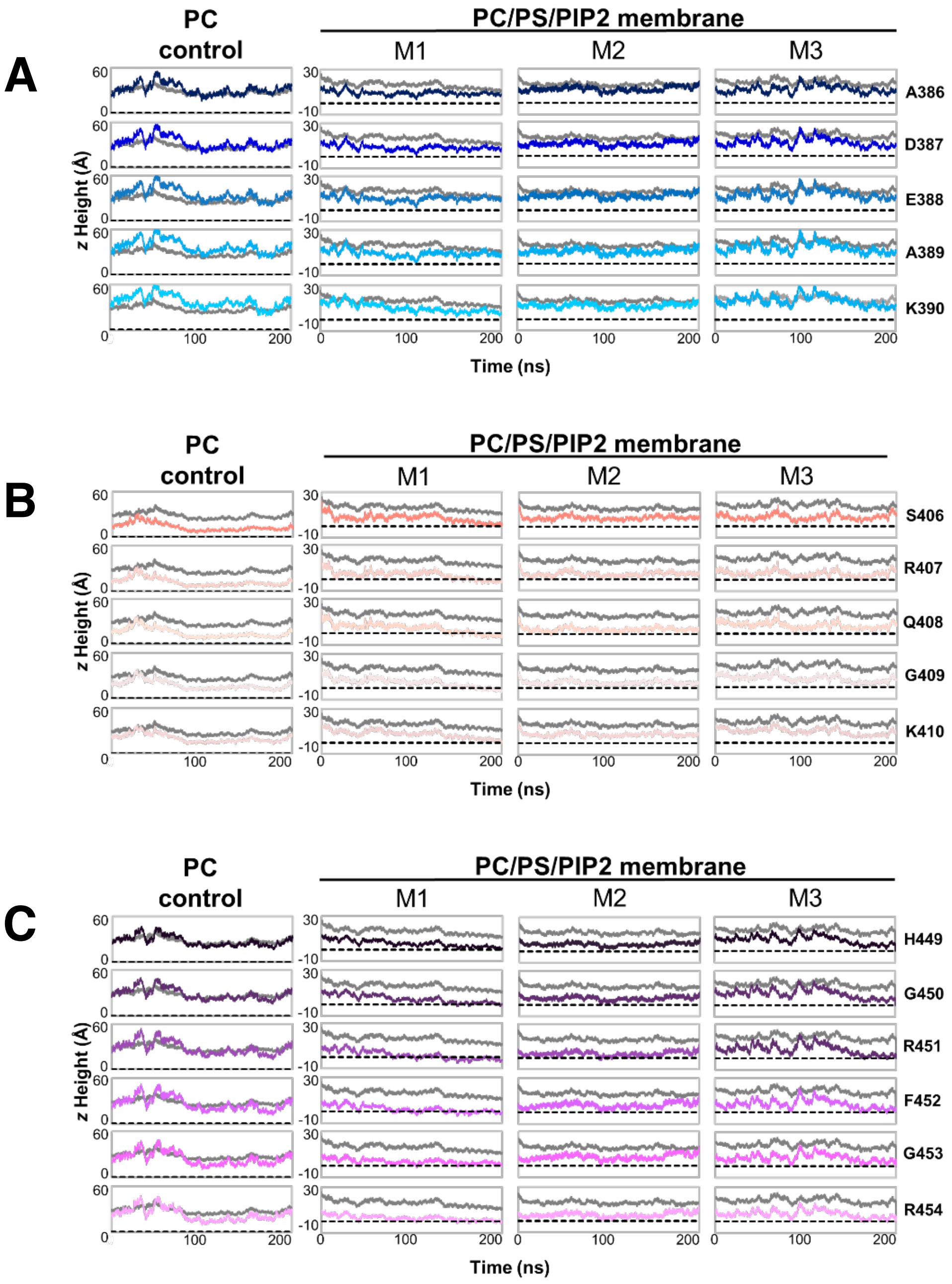

Figure S6. Residue-level penetration calculations. Distances of α-carbons from the membrane phosphate average position (black dashed line) are shown of individual residues over simulation time, with the protein center of mass (gray solid) for reference. Positive values are above the phosphate plane, while negative values indicate insertion. **A:** The β2-β3 loop showed no α-carbon penetration below the phosphate plane in any simulation. **B:** The β3-β4 loop had some penetration near the end of Model 1, and also had the closest contact of the three loops in the PC control. **C:** In the β6-β7 loop, the α-carbons of R451 and F452 penetrated near the membrane phosphate plane in all three PC/PS/PIP_2 models.

Figure S7

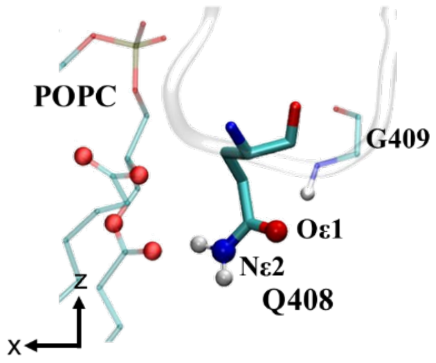

Figure S7. Q408 sidechain H-bonding in Model 1. The polar sidechain of Q408 (thick lines) projected toward the membrane during this simulation, and at times inserted below the phosphate plane. This insertion was stabilized through hydrogen bonding of Nε2 on Q408 (blue sphere) the ester carbonyl oxygen of a nearby POPC lipid (shown as thin lines with carbonyl oxygens as red spheres). At the same time, the arched conformation allowed Oε1 of Q408 (red sphere) to hydrogen bond with the backbone NH of G409 (shown as thin lines with the NH hydrogen as a white sphere).

Figure S8

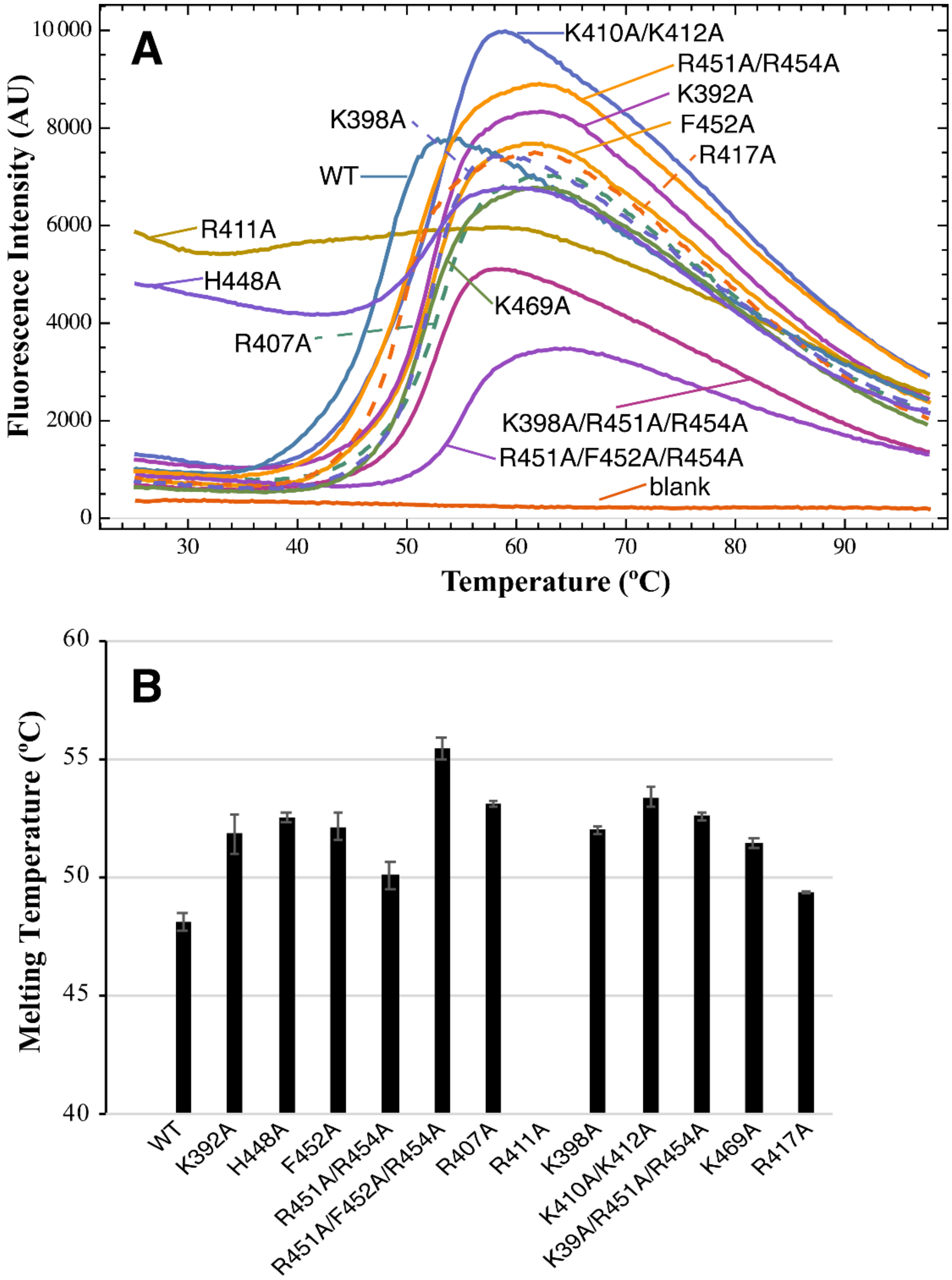

Figure S8: Thermal stability of Slp-4 C2A mutants. Thermal stability was measured using differential scanning fluorimetry, using a StepOne qPCR machine with the protein thermal shift dye kit from Applied Biosystems, and using 0.1 mg/ml protein. Melting temperatures were calculated as the temperature at which the slope of the melt curve was maximal. **A:** Representative melt curves for the wild-type (WT) domain and indicated mutants. **B:** Average ± S.D. from n = 4 replicate measurements. R411A did not have a clear melting transition.

Table S5. PS and PIP_2 contact for mutants compared to WT. Lipid contacts for each indicated mutant were averaged over the last 100 ns of each simulation. Two trajectories each (Sim1 and Sim2) were run for the triple mutants, differing only in a random seed. Blue coloring indicates increased contact relative to WT, while red coloring indicates decreased contact.

		WT		K398A		F452A		K398A/R451A/R454A Sim1		K398A/R451A/R454A Sim2		R451A/F452A/R454A Sim1		R451A/F452A/R454A Sim2	
	**Residue**	Avg PS Contact	Avg PIP_2 Contact	Avg PS Contact	Avg PIP_2 Contact	Avg PS Contact	Avg PIP_2 Contact	Avg PS Contact	Avg PIP_2 Contact	Avg PS Contact	Avg PIP2 Contact	Avg PS Contact	Avg PIP_2 Contact	Avg PS Contact	Avg PIP2 Contact
**β2- β3 loop**	**K390**	0.0 ± 0.1	0	2.0 ± 0.8	0	1.5 ± 0.5	0	0.0 ± 0.0	0	1.3 ± 1.0	0	0.3 ± 0.7	0	0.0 ± 0.0	0
	**K391**	0	0	0	0	0.4 ± 0.5	0	0	0	0	0	0	0	0	0
	**R392**	0.5 ± 0.7	0	1.6 ± 0.7	0	0	0	0.0 ± 0.0	0	1.6 ± 1.1	0	0.3 ± 0.6	0	0.0 ± 0.1	0
**β6- β7 loop**	**H449**	0.0 ± 0.1	0.9 ± 0.3	0	0	0	0	0	0	0	0	0	0	0	0
	**R451/A451**	0.5 ± 0.8	0	1.5 ± 0.6	0	1.0 ± 0.0	0	0.0 ± 0.1	0	0.3 ± 0.4	0	0.4 ± 0.5	0	0.0 ± 0.2	0.0 ± 0.1
	**R454/A454**	0.1 ± 0.3	0.1 ± 0.3	0	0	0	0.0 ± 0.2	0.1 ± 0.3	0.0 ± 0.0	0.0 ± 0.0	0.1 ± 0.2	0.2 ± 0.4	0.0 ± 0.0	0.0 ± 0.2	0.3 ± 0.5
**β3- β4 loop**	**K405**	0.3 ± 0.5	0	0.1 ± 0.3	0	0.0 ± 0.1	0	1.0 ± 0.4	0	0.5 ± 0.5	0	0.2 ± 0.4	0.1 ± 0.2	0.2 ± 0.4	0
	**R407**	0.9 ± 0.5	0	0.8 ± 0.7	0	1.0 ± 0.3	0.2 ± 0.4	1.2 ± 0.5	0	0.7 ± 0.5	0	0.4 ± 0.7	0.0 ± 0.1	0.2 ± 0.4	0
	**R411**	0.7 ± 0.5	0	1.1 ± 0.4	0	0	0.0 ± 0.1	0.0 ± 0.0	0.1 ± 0.3	0.5 ± 0.6	0.0 ± 0.2	0.5 ± 0.5	0	1.5 ± 1.0	0
**Lysine cluster**	**K398/A398**	0.6 ± 0.5	1.0 ± 0.1	0	0	0	1.0 ± 0.1	0	0.0 ± 0.1	0	0.1 ± 0.3	0	1.0 ± 0.2	0	1.0 ± 0.0
	**K410**	0.4 ± 0.5	0	0.0 ± 0.2	0	0.1 ± 0.0	0.8 ± 0.4	0	0.9 ± 0.4	0.1 ± 0.3	0.6 ± 0.5	0	1.0 ± 0.1	0.1 ± 0.2	1.0 ± 0.2
	**K412**	0.0 ± 0.1	1.0 ± 0.0	0.1 ± 0.3	0	0	0.8 ± 0.4	0.0 ± 0.0	0.6 ± 0.5	0	1.0 ± 0.0	0.0 ± 0.0	0.1 ± 0.3	0.1 ± 0.2	1.0 ± 0.0
**Other**	**R417**	0.3 ± 0.5	0.0 ± 0.1	0.1 ± 0.3	0	0.2 ± 0.5	0	0.0 ± 0.1	0	0.2 ± 0.4	0	0.5 ± 0.6	0	0.0 ± 0.2	0
	**R429**	0.0 ± 0.0	0	0.0 ± 0.1	0	0	0	0	0	0.0 ± 0.0	0	0.2 ± 0.5	0	0.0 ± 0.0	0
	**R440**	0.0 ± 0.0	0	0	0	0	0	0.4 ± 0.5	0	0.1 ± 0.2	0	0.0 ± 0.1	0	0.0 ± 0.2	0
	**K483**	0.0 ± 0.0	0	0.0 ± 0.1	0.5 ± 0.5	0.0 ± 0.0	0	0.0 ± 0.0	0.0 ± 0.0	0.0 ± 0.2	0	0	0	0	0
	**Total**	**4.2 ± 1.6**	**3.0 ± 0.5**	**7.4 ± 1.6**	**0.5 ± 0.5**	**4.3 ± 0.9**	**2.8 ± 0.7**	**2.7 ± 0.9**	**1.5 ± 0.7**	**5.1 ± 1.9**	**1.7 ± 0.6**	**2.9 ± 1.7**	**2.1 ± 0.5**	**2.2 ± 1.3**	**3.3 ± 0.5**

= significant (at least 1 σ) increase in contact*

= significant (at least 1 σ) decrease in contact*

= moderate (0.5 – 1 σ) increase in contact*

= moderate (0.5 – 1 σ) decrease in contact*

*All mutants compared to WT

Figure S9

kTSIK (k=phosphogluconoyl-lysine) MSMS fragment spectrum, MH+ = 834.3850

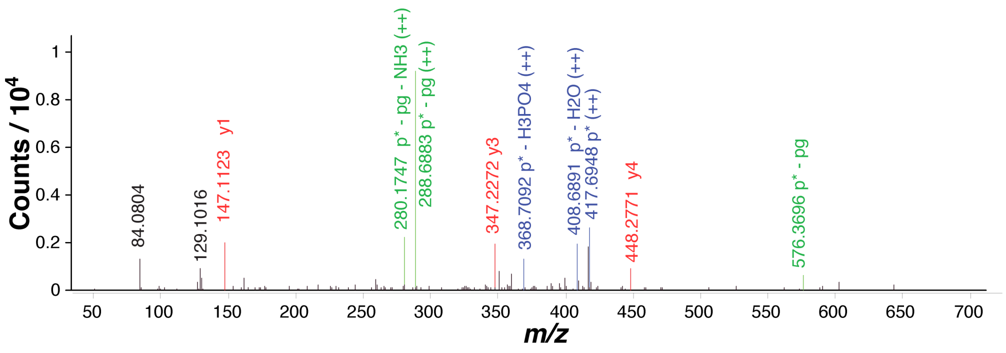

Peak List

Obs. m/z	Pred. m/z	ID
84.0804	84.0808	lysine fragment
129.1016	129.1022	lysine
147.1123	147.1128	y1
280.1747	280.1761	p* - pg - NH3 (++)
288.6883	288.6894	p* - pg (++)
347.2272	347.2289	y3
368.7092	368.7085	p* - H3PO4 (++)
408.6891	408.6911	p*-H2O (++)
417.6948	417.6964	p* (++)
448.2771	448.2766	y4
576.3696	576.3715	p* - pg

KTSIK (unmodified) MSMS fragment spectrum, MH+ = 576.3721

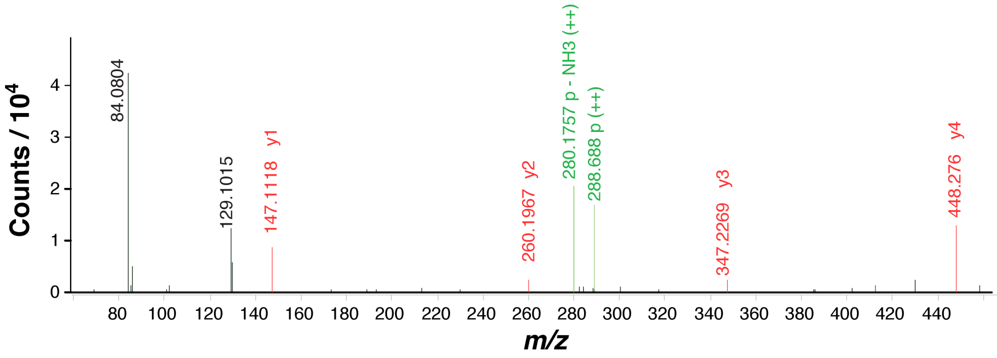

Peak List

Obs. m/z	Pred. m/z	ID
84.0804	84.0808	lysine fragment
129.1015	129.1022	lysine
147.1118	147.1128	y1
260.1967	260.1969	y2
280.1757	280.1761	p - NH3 (++)
288.688	288.6894	p (++)
347.2269	347.2289	y3
448.276	448.2766	y4

Figure S9: Identification of probable phosphogluconoylation at K412. ESI-MS-MS was performed on a tryptic digest of the early peak, and peptides corresponding to modified and unmodified tryptic fragments were identified via molecular formula matching. Shown are MSMS spectra of peptides with the indicated masses (MH+), corresponding to kTSIK$^{412-416}$ (top), where k is phosphogluconoyl-lysine, and KTSIK$^{412-416}$ (bottom). Peaks present in the kTSIK but not KTSIK spectrum correspond to the kTSIK precursor ion (p*) and losses of water and water+phosphate (blue). (Note, the loss of phosphate peak confirms that the phosphate is distal to the gluconoyl group.) Although b-ion fragments are not visible, the y-ion series shows the same pattern in both spectra (red), indicating that the modification is on K412. The KTSIK precursor ion mass (p) is also visible in the kTSIK spectrum consistent with loss of phosphogluconate (p* - pg) (green). ++ indicates $z = 2$.

References:

1. Gurtovenko, A. A., and Vattulainen, I. (2008) Effect of NaCl and KCl on phosphatidylcholine and phosphatidylethanolamine lipid membranes: insight from atomic-scale simulations for understanding salt-induced effects in the plasma membrane. *J Phys Chem B* **112**, 1953-1962
2. Jurkiewicz, P., Cwiklik, L., Vojtiskova, A., Jungwirth, P., and Hof, M. (2012) Structure, dynamics, and hydration of POPC/POPS bilayers suspended in NaCl, KCl, and CsCl solutions. *Biochimica et Biophysica Acta* **1818**, 609-616
3. Seelig, A., and Seelig, J. (1974) The dynamic structure of fatty acyl chains in a phospholipid bilayer measured by deuterium magnetic resonance. *Biochemistry* **13**, 4839-4845
4. Seelig, J., and Waespe-Sarcevic, N. (1978) Molecular order in cis and trans unsaturated phospholipid bilayers. *Biochemistry* **17**, 3310-3315
5. Venable, R. M., Luo, Y., Gawrisch, K., Roux, B., and Pastor, R. W. (2013) Simulations of anionic lipid membranes: development of interaction-specific ion parameters and validation using NMR data. *J Phys Chem B* **117**, 10183-10192
6. Kooijman, E. E., Carter, K. M., van Laar, E. G., Chupin, V., Burger, K. N., and de Kruijff, B. (2005) What makes the bioactive lipids phosphatidic acid and lysophosphatidic acid so special? *Biochemistry* **44**, 17007-17015
7. Wan, C., Wu, B., Song, Z., Zhang, J., Chu, H., Wang, A., Liu, Q., Shi, Y., Li, G., and Wang, J. (2015) Insights into the molecular recognition of the granuphilin C2A domain with PI(4,5)P2. *Chem Phys Lipids* **186**, 61-67
8. Coudevylle, N., Montaville, P., Leonov, A., Zweckstetter, M., and Becker, S. (2008) Structural determinants for Ca^{2+} and phosphatidylinositol 4,5-bisphosphate binding by the C2A domain of rabphilin-3A. *J Biol Chem* **283**, 35918-35928
9. Guerrero-Valero, M., Ferrer-Orta, C., Querol-Audi, J., Marin-Vicente, C., Fita, I., Gomez-Fernandez, J. C., Verdaguer, N., and Corbalan-Garcia, S. (2009) Structural and mechanistic insights into the association of PKCα-C2 domain to PtdIns(4,5)P2. *Proc Natl Acad Sci U S A* **106**, 6603-6607
10. Guillen, J., Ferrer-Orta, C., Buxaderas, M., Perez-Sanchez, D., Guerrero-Valero, M., Luengo-Gil, G., Pous, J., Guerra, P., Gomez-Fernandez, J. C., Verdaguer, N., and Corbalan-Garcia, S. (2013) Structural insights into the Ca^{2+} and PI(4,5)P2 binding modes of the C2 domains of rabphilin 3A and synaptotagmin 1. *Proc Natl Acad Sci U S A* **110**, 20503-20508
